# Catechol-Type Flavonoids from the Branches of *Elaeagnus glabra* f. *oxyphylla* Exert Antioxidant Activity and an Inhibitory Effect on Amyloid-β Aggregation

**DOI:** 10.3390/molecules25214917

**Published:** 2020-10-23

**Authors:** Yu Jin Kim, Eunjin Sohn, Joo-Hwan Kim, MinKyun Na, Soo-Jin Jeong

**Affiliations:** 1 Clinical Medicine Division, Korea Institute of Oriental Medicine, Daejeon 34054, Korea; jinjin0228@kiom.re.kr (Y.J.K.); ssen4022@kiom.re.kr (E.S.); 2 Department of Life Science, Gachon University, Seongnam, Gyeonggi-do 13120, Korea; kimjh2009@gachon.ac.kr; 3 College of Pharmacy, Chungnam National University, Daejeon 34134, Korea; mkna@cnu.ac.kr

**Keywords:** *Elaeagnus glabra* f. *oxyphylla*, Elaeagnaceae, amyloid-β, antioxidant, flavonoid

## Abstract

*Elaeagnus glabra* f. *oxyphylla* (Elaeagnaceae) is a small evergreen tree with narrow lanceolate leaves that is native to Korea. In this work, we studied the chemical composition of *E. glabra* f. *oxyphylla* branches (EGFOB) for the first time. Additionally, we evaluated the effects of the ethanol extract of EGFOB and each of its chemical components on key mediators of Alzheimer’s disease (AD), namely, amyloid-β (Aβ) aggregation and oxidative stress. The ethanol extract of EGFOB decreased Aβ aggregation (IC_50_ = 32.01 µg/mL) and the levels of the oxidative free radicals 2,2′-azino-bis(3-ethylbenzothiazoline-6-sulphonic acid) (ABTS) and 2,2-diphenyl-1-picrylhydrazyl (DPPH) (IC_50_ = 11.35 and 12.32 µg/mL, respectively). Sixteen compounds were isolated from EGFOB. Among them, procyanidin B3 (**8**), procyanidin B4 (**9**), and helichrysoside (**13**) significantly inhibited Aβ aggregation (IC_50_ = 14.59, 32.64, and 44.45 μM, respectively), indicating their potential as bioactive compounds to control Aβ aggregation. Furthermore, these compounds markedly enhanced in vitro scavenging activity against ABTS (IC_50_ = 3.21–4.61 µM). In the DPPH test, they showed lower scavenging activity than in the ABTS test (IC_50_ ≥ 54.88 µM). Thus, these results suggest that EGFOB and specifically compounds **8**, **9**, and **13** may be beneficial in AD prevention and treatment through their antioxidant and anti-Aβ aggregation activities.

## 1. Introduction

Alzheimer’s disease (AD) is a progressive neurodegenerative disorder with no available cure. The incidence rate is higher among people over the age of 60, with AD patients accounting for 60–80% of all dementia cases [1,2]. Currently, the number of dementia patients in the world is estimated at 44 million, and this number is predicted to more than triple by 2050 [3].

AD is characterized by cognitive disruptions, such as memory loss and language difficulties, and non-cognitive dysfunctions, such as behavioral disturbance, depression, hallucination, and delusion [4,5]. The hallmark of AD pathogenesis is the progressive accumulation of amyloid-β (Aβ) in the brain [6]. Aβ plaques are extracellular accumulations of pathological forms of Aβ that are principally composed of abnormally folded Aβ, such as Aβ_1-40_ and Aβ_1–42_. Because of its higher rates of fibrillation and insolubility, Aβ_1–42_ is more abundant in the plaques than Aβ_1-40_ [3]. Aβ aggregation can be initiated by oxidative stress in the early stage of AD, and Aβ plaques also influence free radical generation and oxidative stress [7,8,9]. The excessive generation of free radicals in pathological AD conditions causes an imbalance between free radicals and antioxidants, which are conditions for oxidative stress [9]. Furthermore, previous studies have indicated that Aβ_1–42_ is neurotoxic. It increases the oxidation of proteins in the cell, thereby leading to neuronal dysfunction and cell death during the late stage of AD [10]. The neurotoxicity of abnormal Aβ deposition results from oxidative stress, which is central in the neuronal cell death and inflammation associated with the pathogenesis of AD [11]. It has been reported that antioxidants can attenuate the symptoms of AD and prevent progression [7]. Therefore, new AD drugs that target Aβ with potent antioxidant activity may be useful in the prevention and treatment of AD.

Unfortunately, there is currently no cure for AD, and the current treatment strategies are aimed at symptom relief rather than correction of the underlying cause. There are two classes of medications approved for AD by the US Food and Drug Administration, namely, cholinesterase inhibitors (ChEIs) (donepezil, rivastigmine, and galantamine) and the *N*-methyl-D-aspartate (NMDA) receptor antagonist memantine [9]. These drugs temporarily alleviate symptoms, thereby improving patients’ quality of life, but there is no evidence that they suppress AD progression. In addition, the administration of these drugs has been reported to cause side effects, such as leg cramps, flatulence, and increased gastrointestinal secretions for ChEIs, and confusion, dizziness, constipation, and headache for NMDA receptor antagonists [3,12]. In contrast, most natural products have lower toxicity and fewer side effects [13,14] and thus represent attractive therapeutic candidates.

*Elaeagnus glabra* f. *oxyphylla* (Servett.) W. T. Lee (Elaeagnaceae) is a small evergreen tree with narrower lanceolate leaves than those of *E*. *glabra* [15]. This plant species is native to Korea (Jeju) and is also distributed in China. Several studies have described the pharmacological effects and phytochemicals of plants belonging to the genus *Elaeagnus*. It has been reported that *E*. *glabra* has anti-tumor and antibacterial effects, and it has been used to treat tetanus, asthma, and diarrhea [16,17]. *E*. *angustifolia* exerts antinociceptive, anti-inflammatory, antimicrobial, antioxidant, and antimutagenic activities [18,19]. *E*. *umbellate* is known to have antidiabetic, antioxidant, and anticancer properties [20,21]. Interestingly, our previous study reported the memory-ameliorating effects of *E*. *glabra* f. *oxyphylla* in a mouse model of scopolamine-induced memory impairment [22]. Previous studies have reported that the genus *Elaeagnus* contains flavonoids, triterpenoids, lignan glycosides, and phenolic compounds [17,20,23]. However, there have been no phytochemical studies using *E. glabra* f. *oxyphylla* extracts.

Since the neuronal Aβ aggregation in AD occurs early during the pathogenesis and appears to be the main pathogenic cause [6], blocking Aβ accumulation may suppress the progression of AD [24]. Therefore, in the present study, we assessed ethanol extracts of *E. glabra* f. *oxyphylla* branches (EGFOB) and leaves (EGFOL) and their chemical components for their inhibitory effects on Aβ aggregation.

## 2. Results and Discussion

### 2.1. Inhibitory Effects of EGFOB and EGFOL on Aβ Aggregation

**Figure 1:**
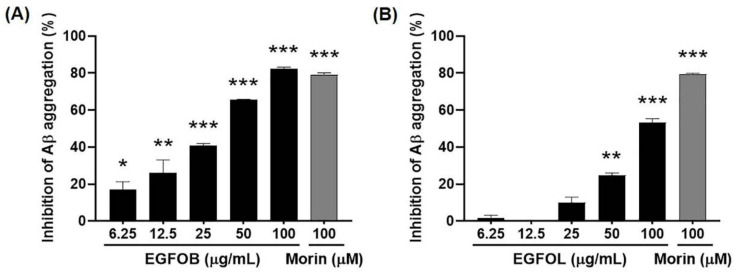
Inhibitory effects of *E. glabra* f. *oxyphylla* branches (EGFOB) and leaves (EGFOL) extracts on amyloid-β (Aβ) aggregation. Various concentrations (6.25, 12.5, 25, 50, or 100 μg/mL) of (**A**) EGFOB and (**B**) EGFOL were prepared and reacted with Aβ_1–42_ peptides, followed by the addition of Thioflavin (ThT). Fluorescence intensity was measured at 440 nm (excitation) and 485 nm (emission). Each value is expressed as the mean ± SEM (*n* = 3). ^*^p < 0.05, ^**^p < 0.01, or ^***^p < 0.001 vs. control.

To explore whether *E. glabra* f. *oxyphylla* has an inhibitory effect on Aβ aggregation, we prepared ethanol extracts of EGFOB and EGFOL at various concentrations (6.25, 12.5, 25, 50, or 100 µg/mL). Both EGFOB and EGFOL extracts inhibited in vitro Aβ aggregation in a dose-dependent manner (IC_50_ = 32.01 and 92.97 µg/mL, respectively) (Figure 1A, B). These results indicate that EGFOB have a stronger inhibitory effect on Aβ aggregation compared with EGFOL. Aβ aggregation is one of the primary pathological indicators in AD patients [6]. Despite continuous debate about the relevance of Aβ as a target biomarker in new drug development for AD, many researchers are still focusing on Aβ [25,26,27]. The amyloid hypothesis suggests that the abnormality of Aβ is observed somewhat earlier before the onset of AD compared with other AD biomarkers [28]. Therefore, Aβ should be considered an important priority in investigations for the prevention as well as the treatment of AD. In our study, the robust activity of EGFOB is a notable result for anti-Aβ therapy research.

### 2.2. Antioxidant Effects of EGFOB and EGFOL

**Figure 2:**
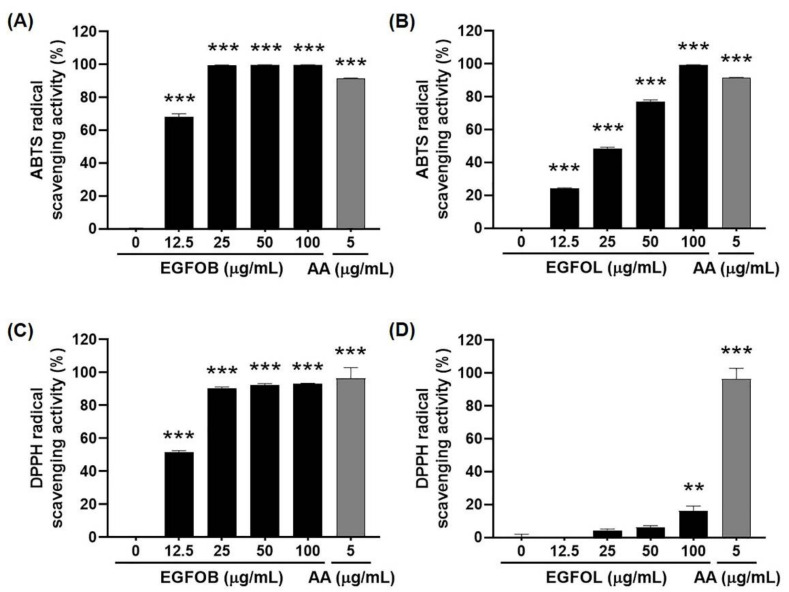
Antioxidant effects of *E. glabra* f. *oxyphylla* branches (EGFOB) and leaves (EGFOL). Antioxidant activity was measured by free radical scavenging assay for 2,2′-azino-bis(3-ethylbenzothiazoline-6-sulphonic acid) (ABTS) (**A**,**B**) and 2,2-diphenyl-1-picrylhydrazyl (DPPH) (**C**,**D**). Each value is presented as the mean ± SEM (*n* = 3). Ascorbic acid (AA) was used as a positive control. ^**^p < 0.01 or ^***^p < 0.001 vs. control.

Oxidative stress induced by Aβ in the brain is related to the pathogenesis of AD [11]. Previous studies have reported that many natural products have robust antioxidant properties [29,30]. To examine the antioxidant activities of EGFOB and EGFOL, 2,2′-azino-bis(3-ethylbenzothiazoline-6-sulphonic acid) (ABTS) and 2,2-diphenyl-1-picrylhydrazyl (DPPH) radical scavenging assays, widely used to assess the antioxidant efficacy of natural products [29,30], were performed. The EGFOB and EGFOL extracts reached 100% ABTS radical scavenging activity at the concentrations of 25 and 100 µg/mL, respectively (IC_50_ = 11.35 and 26.01 µg/mL, respectively) (Figure 2A,B). While the EGFOB extract showed similar results in the DPPH assay to those in the ABTS assay, the EGFOL extract had a significant effect on DPPH radical scavenging only at 100 µg/mL (IC_50_ = 12.32 and >100 µg/mL, respectively) (Figure 2C,D). The remarkable radical scavenging activity of EGFOB extract against ABTS and DPPH indicates the antioxidant capacity of EGFOB. Previous studies have been reported focusing on the relationship between free radicals and the formation of Aβ aggregates in AD development. Aβ plaques contributes to the generation of free radicals and oxidative stress [31], and it was found that oxidative stress is closely related to Aβ aggregation [32]. As is well known, there are many natural products that are powerful antioxidants and can play an important role in the regulation of various chronic diseases, including AD. In new drug development for AD, natural extracts such as EGFOB are attractive agents with dual- or multi-function. By comparing the effects of the two extracts in the Aβ aggregation and radical scavenging assays according to the IC_50_ values, it was found that the EGFOB extract was more effective than the EGFOL extract.

### 2.3. Inhibitory Effects of the Solvent Fractions of EGFOB on Aβ Aggregation

**Figure 3:**
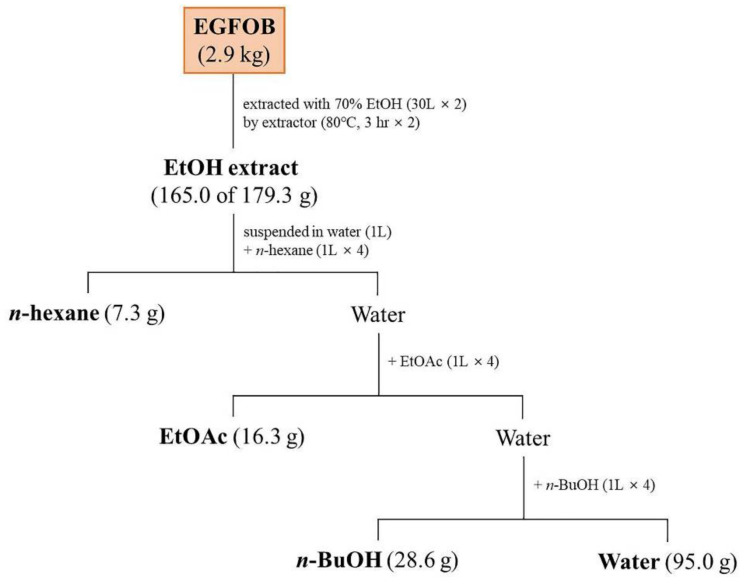
Extraction and fractionation of *E. glabra* f. *oxyphylla* branches (EGFOB).

**Figure 4:**
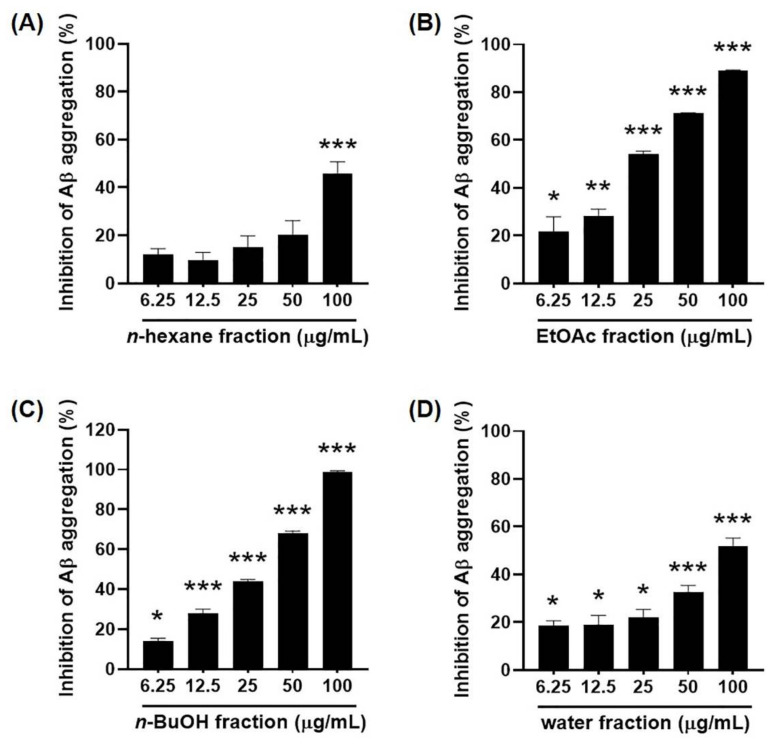
Inhibitory effects of solvent fractions of EGFOB on amyloid-β (Aβ) aggregation. Various concentrations (6.25, 12.5, 25, 50, or 100 μg/mL) of (**A**) *n*-hexane, (**B**) EtOAc, (**C**) *n*-BuOH, and (**D**) water fractions were prepared and reacted with Aβ_1–42_ peptides, followed by the addition of ThT. Fluorescence intensity was measured at 440 nm (excitation) and 485 nm (emission). Each value is expressed as the mean ± SEM (*n* = 3). ^*^p < 0.05, ^**^p < 0.01, or ^***^p < 0.001 vs. control.

The ethanol extract of EGFOB was suspended in water and successively fractionated by using *n*-hexane, ethyl acetate (EtOAc), and *n*-butanol (*n*-BuOH) (Figure 3). The Aβ aggregation assay was performed using the water, *n*-hexane, EtOAc, and *n*-BuOH fractions. As shown in Figure 4, the EtOAc- and *n*-BuOH-soluble fractions significantly inhibited Aβ aggregation (IC_50_ = 24.31 and 30.24 µg/mL, respectively), unlike the other fractions. This observation indicates that the EtOAc- and *n*-BuOH-soluble fractions contain the bioactive compounds of EGFOB against Aβ aggregation.

### 2.4. Isolation and Identification of Compounds 1–16 from EGFOB

**Figure 5:**
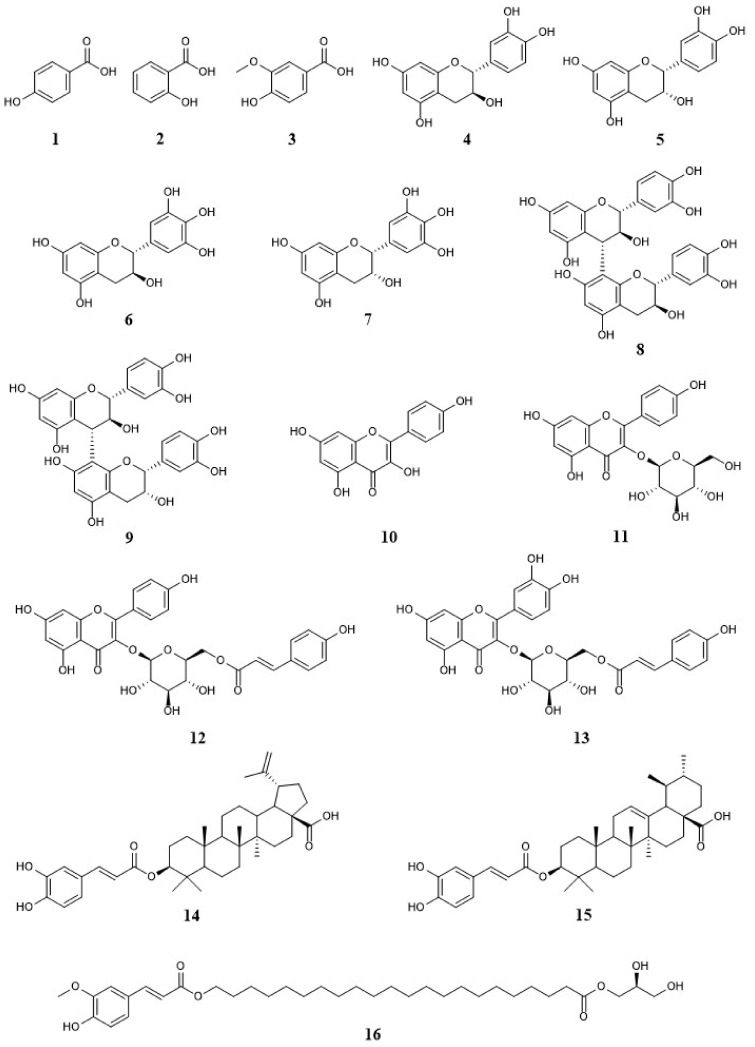
Structures of compounds **1**–**16** constituting EGFOB. 4-hydroxybenzoic acid (**1**), salicylic acid (**2**), vanillic acid (**3**), (+)-catechin (**4**), (–)-epicatechin (**5**), (+)-gallocatechin (**6**), (–)-epigallocatechin (**7**), procyanidin B3 (**8**), procyanidin B4 (**9**), kaempferol (**10**), astragalin (**11**), *trans*-tiliroside (**12**), helichrysoside (**13**), betulinic acid-3-*O*-*trans*-caffeate (**14**), ursolic acid-3-*O*-*trans*-caffeate (**15**), and 1-mono(22-*O*-feruloyl-oxydocosanoyl)glycerol (**16**).

To identify the chemical components that have antioxidative activities and inhibit Aβ aggregation, we isolated the chemical constituents from the EtOAc-soluble fraction of EGFOB, which significantly inhibited Aβ aggregation and had more components than BuOH-soluble fraction according to the HPLC chromatogram (data not shown). We further fractionated the EtOAc-soluble fraction by using silica gel column chromatography to produce six subfractions (F1–F6). F1–F4 were further resolved using octadecylsilane (ODS) and Sephadex LH-20 column chromatography and preparative reversed-phase HPLC. Finally, sixteen compounds (**1**–**16**), including three phenols (**1**–**3**), ten flavonoids (**4**–**13**), two triterpenoids (**14**, **15**), and one fatty acid ester (**16**), were purified (Figure 5). By comparing the spectral and physicochemical properties of these compounds (Supplementary Materials) with those of previously reported compounds, the sixteen compounds were identified as 4-hydroxybenzoic acid (**1**) [33], salicylic acid (**2**) [34], vanillic acid (**3**) [35], (+)-catechin (**4**) [36], (–)-epicatechin (**5**) [37], (+)-gallocatechin (**6**) [36], (–)-epigallocatechin (**7**) [38], procyanidin B3 (**8**) [39], procyanidin B4 (**9**) [40], kaempferol (**10**) [41], astragalin (**11**) [42], *trans*-tiliroside (**12**) [38], helichrysoside (**13**) [43], betulinic acid-3-*O*-*trans*-caffeate (**14**) [44], ursolic acid-3-*O*-*trans*-caffeate (**15**) [45], and 1-mono(22-*O*-feruloyl-oxydocosanoyl)glycerol (**16**) [46]. Previous studies have reported the presence of various types of phytochemicals in the genus *Elaeagnus*, including flavonoids (e.g., rutin, epigallocatechin gallate, and isorhamnetin) and phenolic compounds (e.g., chlorogenic acid, pyrogallol, and ferulic acid) from *E. umbellata* [20], *E. angustifolia* [47], and *E. pungens* [48]; and triterpenoid saponins (e.g., terpengustifol), lignan glycosides (e.g., phengustifols A and B), and alkaloids (e.g., harmane and tetrahydroharmol) from *E. angustifolia* [23,47]. Of note, this study represents for the first time these sixteen compounds have been identified in EGFOB.

**Figure 6:**
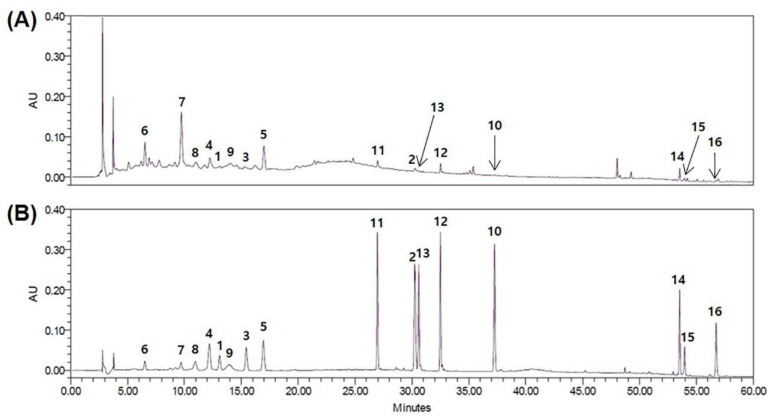
HPLC chromatograms of the ethanol extract of (**A**) EGFOB and (**B**) isolated compound mixture at 240 nm.

The isolated compounds were simultaneously analyzed using HPLC. The sixteen compounds were successfully separated on a Luna Omega C_18_ analytical column (4.6 × 250 mm, 5 µm, Phenomenex) at 30 °C with two mobile phases consisting of 0.1% (*v*/*v*) aqueous trifluoroacetic acid (TFA) (a) and acetonitrile (ACN) (b) within 60 min. The conditions of the gradients were 10–15% (b) for 0–15 min, 15–50% (b) for 15–40 min, 50–100% (b) for 40–50 min, and 100% (b) for 50–60 min. The HPLC chromatograms of the ethanol extract of EGFOB and isolated compound mixture at 240 nm are presented in Figure 6.

### 2.5. Inhibitory Effects of Compounds 1–16 from EGFOB on Aβ Aggregation

**Figure 7:**
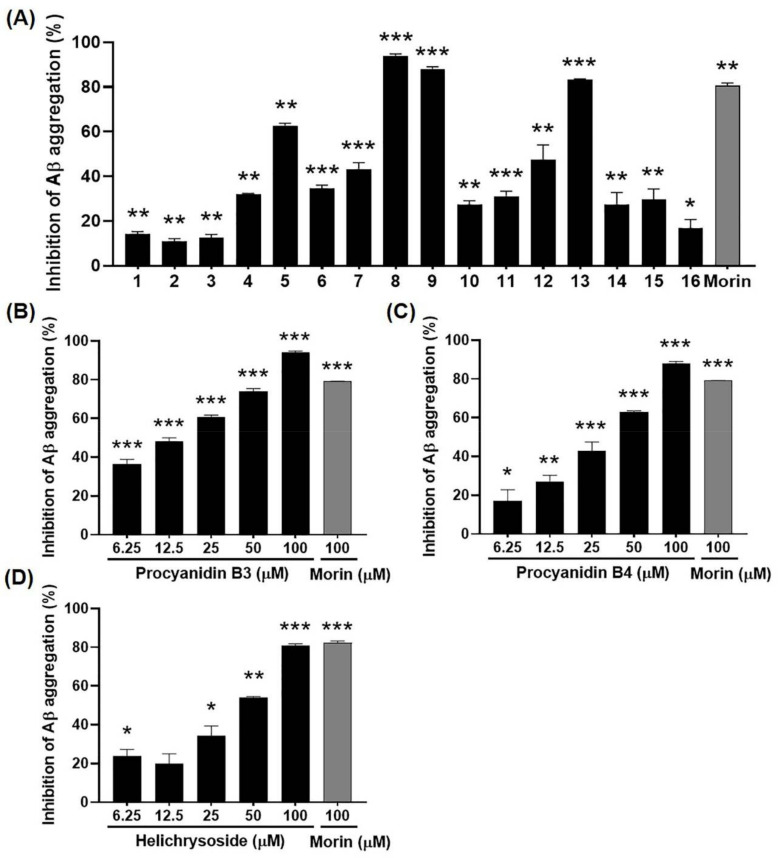
Inhibitory effects of compounds **1**–**16** from EGFOB on amyloid-β (Aβ) aggregation. Compounds **1**–**16** (100 μM) isolated from EGFOB (**A**) and various concentrations (6.25, 12.5, 25, 50, or 100 μM) of procyanidin B3 (**8**) (**B**), procyanidin B4 (**9**) (**C**), and helichrysoside (**13**) (**D**) were reacted with Aβ_1–42_ peptides, followed by the addition of ThT. Fluorescence was measured at 440 nm (excitation) and 485 nm (emission). Morin (100 μM) was used as a positive control. Each value is shown as the mean ± SEM (*n* = 3). ^*^p < 0.05, ^**^p < 0.01, or ^***^p < 0.001 vs. control.

We evaluated the biological activities of the sixteen compounds through an in vitro Aβ aggregation assay and a free radical scavenging assay. Compounds **8**, **9**, and **13** inhibited Aβ aggregation comparably to morin, a known inhibitor of Aβ aggregation [49] (Figure 7A). Subsequently, we evaluated the inhibitory effects of these compounds on Aβ aggregation at various concentrations (6.25, 12.5, 25, 50, or 100 µM). Compounds **8**, **9**, and **13** significantly inhibited Aβ aggregation in a dose-dependent manner (IC_50_ = 14.59, 32.64, and 44.45 μM, respectively) (Figure 7B–D). These results indicate the potential of compounds **8**, **9**, and **13** as bioactive compounds to control Aβ aggregation. The inhibitory rates of the other thirteen compounds were <63%.

It has been reported that phenolic compounds, including flavonoids, prevent oligomerization of Aβ by changing its conformation [50]. Structure–activity relationship studies have revealed that the catechol moiety on ring B of flavonoids plays a critical role in their inhibitory activities against Aβ aggregation, and catechol-type flavonoids can specifically inhibit Aβ_1–42_ aggregation by targeting lysine residues. In addition, flavonols containing a C2–C3 double bond on ring C have been found to inhibit Aβ_1–42_ aggregation by reacting with the β-sheet regions of Aβ_1–42_ aggregates [51,52]. These findings are in line with our observation that compounds **8** (catechin-(4α→8)-catechin dimer), **9** (catechin-(4α→8)-epicatechin dimer), and **13** (catechol-type flavonol) had stronger inhibitory effects on Aβ aggregation than other compounds.

### 2.6. Antioxidant Effects of Compounds 1–16 from EGFOB

**Figure 8:**
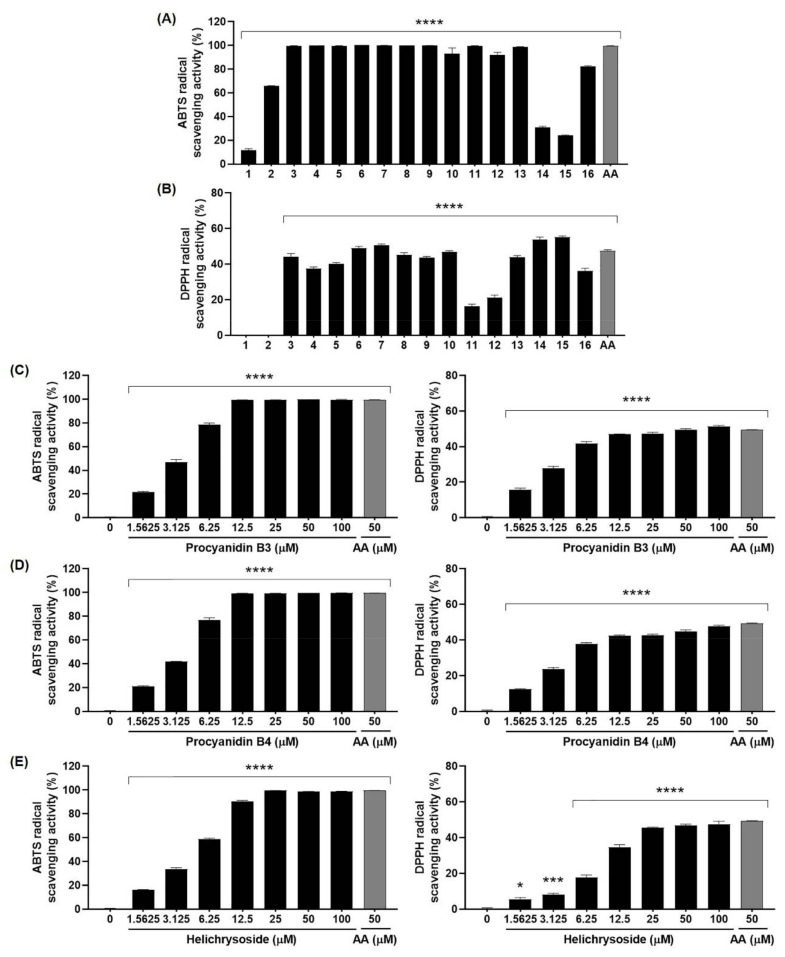
Antioxidant effects of compounds **1**–**16** from EGFOB. Antioxidant activities of compounds **1**–**16** (100 μM) (**A**,**B**), procyanidin B3 (**8**) (**C**), procyanidin B4 (**9**) (**D**), and helichrysoside (**13**) (**E**) (1.5625–100 μM) were measured using ABTS and DPPH radical scavenging assays. Ascorbic acid (AA) (50 μM) was used as a positive control. Each value is presented as the mean ± SEM (*n* = 3). ^*^p < 0.05, ^***^p < 0.001, or ^****^p < 0.0001 vs. control.

To examine the antioxidant activities of the sixteen compounds isolated from EGFOB, ABTS and DPPH radical scavenging assays were performed. Twelve compounds, excluding compounds **1**, **2**, **14**, and **15**, significantly increased radical scavenging activity against ABTS at 100 μM (Figure 8A). The scavenging activities of compounds **1**, **2**, **14**, and **15** against ABTS were <66%. However, the DPPH radical scavenging activities of the sixteen compounds were <56% (Figure 8B). Additionally, we evaluated the ABTS and DPPH radical scavenging activities of compounds **8**, **9**, and **13**, candidate inhibitors of Aβ aggregation, at various concentrations from 1.5625 to 100 µM. As shown in Figure 8C–E, compounds **8** (IC_50_ = 3.21 µM), **9** (IC_50_ = 3.44 µM), and **13** (IC_50_ = 4.61 µM) dose-dependently enhanced radical scavenging activity against ABTS, which reached 100% at 12.5, 12.5, and 25 µM, respectively. In the DPPH test, we observed that the scavenging activity patterns of compounds **8**, **9**, and **13** were similar to those detected in the ABTS test. However, the DPPH radical scavenging activities of these three compounds were <52% (IC_50_ = 54.88, >100, and >100 µM, respectively). As mentioned above, since Aβ aggregation and free radicals are closely related, compounds **8**, **9**, and **13** targeting Aβ with potent antioxidant activity are promising as valuable candidates for the prevention and treatment of AD. Additional studies will be necessary to identify the bioactive compounds of EGFOB in the inhibition of AD pathogenesis. In vitro and in vivo assays are required to determine the efficacy and safety of each candidate bioactive compound.

## 3. Materials and Methods

### 3.1. General Experimental Procedures

^1^H-NMR (500 MHz), ^13^C-NMR (125 MHz), and 2D NMR (HMQC, HMBC, and COSY) experiments were conducted on a Bruker Avance 500 NMR spectrometer (Bruker, Billerica, MA, USA). HRESI-MS and ESI-MS data were recorded on a SYNAPT G2 (Waters, Milford, MA, USA) mass spectrometer and Waters Acquity UPLC H-class system equipped with a QDa detector (Waters, Waters, Milford, MA, USA), respectively. Optical rotations were measured using the JASCO P-2000 digital polarimeter (JASCO, Tokyo, Japan). Infrared (IR) spectra were recorded on a Bruker ALPHA-T IR Spectrometer (Bruker, Billerica, MA, USA). Melting points were measured using a Mettler Toledo^TM^ MP50 melting point apparatus (Mettler Toledo, Columbus, OH, USA). Column chromatography was performed using silica gel (70–230 mesh, Merck, Darmstadt, Germany), ODS-A (S-75 µm, YMC Co., Ltd., Kyoto, Japan), and Sephadex^TM^ LH-20 (GE Healthcare, Chicago, IL, USA). Preparative HPLC was performed on Luna C_18_ (21.2 × 250 mm, 5 µm, Phenomenex, Torrance, CA, USA), Triart C_18_ (20 × 250 mm, 5 µm, YMC Co., Ltd., Kyoto, Japan), and Luna C_18_ (10 × 250 mm, 5 µm, Phenomenex, Torrance, CA, USA) columns with analytical grade water and ACN (J. T. Baker Chemical Co., Phillipsburg, NJ, USA) as mobile phases by using the Interface LC 3000 system. Thin-layer chromatography was performed on 60 F_254_ or RP-18 F_254_ (Merck, Darmstadt, Germany) pre-coated with silica gel. To identify the isolated compounds from their HPLC chromatogram, HPLC was performed on a Luna Omega C_18_ (4.6 × 250 mm, 5 µm, Phenomenex, Torrance, CA, USA) analytical column by using a Waters Alliance e2695 system equipped with a photodiode array detector (#2998, Waters, Milford, MA, USA). The analytical grade reagent TFA was purchased from Sigma-Aldrich (St. Louis, MO, USA).

### 3.2. Plant Materials

Dried EGFOB and ethanol extracts of EGFOL were obtained from the Korean Seed Association. The origins of the herbal materials were identified by Professor Joo-Hwan Kim (Gachon University, Seongnam, Korea). A voucher specimen (SCD-A-112) was deposited at the Clinical Medicine Division of Korea Institute of Oriental Medicine (Daejeon, Korea).

### 3.3. Extraction, Fractionation, and Isolation

The dried EGFOB (2.9 kg) were extracted twice with 70% aqueous ethanol (30 L each time) by using an electric extractor (COSMOS-660, Kyungseo Machine Co., Incheon, Korea) at 80 ± 2 °C for 3 h. The extract solution was filtered, concentrated using a rotary evaporator system (EYELA N-12, Rikakikai Co., Tokyo, Japan) under vacuum, and freeze-dried to obtain a powdered extract (179.3 g, 6.2%). This powdered extract (165.0 g) was suspended in water (1 L) and successively partitioned four times with 1 L each of *n*-hexane, EtOAc, and *n*-BuOH to obtain *n*-hexane (7.3 g), EtOAc (16.3 g), and *n*-BuOH (28.6 g)-soluble fractions. The EtOAc-soluble fraction (15.5 g), which significantly inhibited Aβ aggregation, was subjected to silica gel column chromatography (70–230 mesh, 11 × 19 cm) and eluted with a stepwise gradient of CH_2_Cl_2_–MeOH (100:1, 80:1, 40:1, 20:1, 10:1, 5:1, 3:1, 2:1, 1:1, and 0:1) to afford six fractions (F1–F6). F1 (960.0 mg) was subjected to ODS column chromatography (S-75 µm, 2.3 × 40 cm) and eluted with an MeOH–H_2_O gradient (60:40→100:0) to obtain ten fractions (F1-1–F1-10). F1-8 (79.0 mg) was further purified using preparative HPLC with a Luna C_18_ column (21.2 × 250 mm, 5 µm) containing ACN–H_2_O (94:6) to obtain compound **16** (12.4 mg). F2 (746.0 mg) was chromatographed on the ODS column (2.3 × 40 cm) and eluted with an MeOH–H_2_O gradient (40:60→100:0) to obtain nine fractions (F2-1–F2-9). F2-4 (65.7 mg) was subjected to preparative HPLC (21.2 × 250 mm, 5 µm, Luna C_18_) with ACN–H_2_O (45:55) as the eluent and then recrystallized to obtain compound **10** (3.8 mg). Compound **15** (28.3 mg) was isolated from F2-8 (117.0 mg) by preparative HPLC (21.2 × 250 mm, 5 µm, Luna C_18_) with ACN–H_2_O (88:12) as the eluent. F3 (5.1 g) was chromatographed on an ODS column (4 × 49 cm) and eluted with an MeOH–H_2_O gradient (10:90→100:0) to obtain twenty-four fractions (F3-1–F3-24). Compound **1** (10.0 mg) was isolated from F3-1 (90.0 mg) through silica gel column (2.3 × 42 cm) chromatography involving a CH_2_Cl_2_–MeOH gradient (10:1→1:1) as the eluent. F3-5 (1.3 g) was subjected to silica gel column (3 × 45 cm) chromatography and eluted with a CH_2_Cl_2_–MeOH gradient (40:1→1:1) to obtain compound **4** (250.0 mg). Subfraction F3-5-6 (217.0 mg) was further purified using preparative HPLC (20 × 250 mm, 5 µm, Triart C_18_) with ACN–H_2_O (13:87) as the eluent to obtain compounds **7** (39.5 mg) and **9** (27.0 mg). F3-6 (226.0 mg) was chromatographed on a silica gel column (2.3 × 32 cm) by eluting with a CH_2_Cl_2_–MeOH gradient (40:1→1:1) and further purified using preparative HPLC (20 × 250 mm, 5 µm, Triart C_18_) with ACN–H_2_O (8:92) as the eluent to obtain compound **3** (4.6 mg). F3-7 (534.0 mg) was subjected to consecutive preparative HPLC (20 × 250 mm, 5 µm, Triart C_18_) to obtain compound **5** (68.0 mg). Compounds **11** (18.0 mg) and **12** (30.0 mg) were isolated from F3-15 (100.0 mg) and F3-17 (178.0 mg), respectively, by using preparative HPLC (10 × 250 mm, 5 µm, Luna C_18_) with ACN–H_2_O (19:81) as the eluent, followed by recrystallization. Compound **14** (13.5 mg) was purified from F3-24 (70.0 mg) by using preparative HPLC (21.2 × 250 mm, 5 µm, Luna C_18_) with an ACN–H_2_O gradient (83:17→84:16) as the eluent. F4 (4.4 g) was applied on a Sephadex LH-20 column (4.2 × 50 cm) and eluted with an MeOH–H_2_O gradient (10:90→100:0) to obtain ten fractions (F4-1–F4-10). Compound **2** (20.0 mg) was isolated from F4-2 (72.0 mg) by using preparative HPLC (21.2 × 250 mm, 5 µm, Luna C_18_) with ACN–H_2_O (2:98) as the eluent. F4-5 (285.5 mg) was chromatographed on a silica gel column (2.2 × 50 cm) with a CH_2_Cl_2_–MeOH gradient (40:1→1:1) as the eluent and subsequently purified through preparative HPLC (21.2 × 250 mm, 5 µm, Luna C_18_, ACN–H_2_O (6:94)) to obtain compound **6** (7.0 mg). F4-6 (1.4 g) was subjected to silica gel column (3.2 × 40 cm) chromatography involving a CH_2_Cl_2_–MeOH gradient (40:1→1:1) as the eluent and purified through consecutive preparative HPLC (21.2 × 250 mm, 5 µm, Luna C_18_) to obtain compound **8** (13.5 mg). Compound **13** (3.6 mg) was isolated from F4-9 (262.0 mg) through consecutive preparative HPLC (21.2 × 250 mm, 5 µm, Luna C_18_) with ACN–H_2_O (27:73) and ACN–H_2_O (24:76) as the eluents.

### 3.4. Aβ Aggregation Assay

To measure the inhibitory effect on Aβ_1–42_ aggregation, the SensoLyte^®^ Thioflavin T (ThT) β-Amyloid aggregation kit (AnaSpec, Fremont, CA, USA) was used according to the modified manufacturer’s instructions. This assay is based on the property of ThT dye that increases fluorescence when bound to the aggregates of Aβ_1–42_ peptides. The detailed protocol is described in a previous report [53]. Morin (100 μM) was used as a positive control for the inhibition of Aβ aggregation. Experiments were performed in triplicate and independently repeated three times. The inhibition rate (%) of Aβ aggregation was calculated according to the following equation:

(1) $$\mathrm{Inhibition}\;\mathrm{of}\;A\beta \;\mathrm{aggregation}\;\left(\%\right)=\left(1-\frac{\mathrm{Fluorescence}\;\mathrm{of}\;A\beta -\mathrm{treated}\;\mathrm{sample}}{\mathrm{Fluorescence}\;\mathrm{of}\;\mathrm{untreated}\;\mathrm{sample}}\right)\times 100$$

### 3.5. Free Radical Scavenging Assay

ABTS and DPPH radical scavenging assays were performed with reference to the modified method described in previously published reports [54,55] to examine the antioxidant activities. In the ABTS assay, 7 mM ABTS aqueous solution and 2.45 mM potassium persulfate were reacted for 12–16 h in the dark at room temperature to prepare the ABTS^•+^ solution. Subsequently, the reactant was diluted 25-fold to adjust its absorbance at 734 nm to 0.7 by using a spectrophotometer (Benchmark Plus, Bio-Rad, Hercules, CA). The sample solution (100 μL) and ABTS^•+^ solution (100 μL) were mixed and incubated for 5 min at room temperature in the dark.

To determine the DPPH radical scavenging activity, the sample solution (100 μL) and 0.15 mM DPPH solution (100 μL) were mixed and incubated for 3 h at room temperature in the dark. The absorbance was measured at 517 nm. Ascorbic acid was used as a positive control for antioxidative activity. The radical scavenging activity (%) was calculated using the following equation:

(2) $$\mathrm{Radical}\;\mathrm{scavenging}\;\mathrm{activity}\;\left(\%\right)=\left(1-\frac{\mathrm{Absorbance}\;\mathrm{of}\;\mathrm{sample}}{\mathrm{Absorbance}\;\mathrm{of}\;\mathrm{control}}\right)\;\times \;100$$

### 3.6. Statistical Analysis

Calculated values were expressed as mean ± SEM. One-way analysis of variance or Student’s *t*-test was performed using GraphPad Prism 7.0 (GraphPad Software, San Diego, CA, USA) to determine statistical significance. *P*-values <0.05 were considered statistically significant.

## 4. Conclusions

Our data demonstrate that the ethanol extract of EGFOB has anti-Aβ aggregation and antioxidant activities. Sixteen compounds (**1**–**16**) were isolated from the ethanol extract of EGFOB by using chromatographic techniques. The chemical structures of these compounds were determined using 1D and 2D NMR spectroscopy, or electrospray ionization mass spectrometry. Among these sixteen compounds, compounds **8**, **9**, and **13** showed potent antioxidative and anti-Aβ aggregation activities. These results suggest that EGFOB, especially compounds procyanidin B3 (**8**), procyanidin B4 (**9**), and helichrysoside (**13**), may be promising candidate therapeutics against AD and AD-related diseases.

## Supplementary Materials

The NMR spectra (Figures S1–S33), spectroscopic and physicochemical data of compounds **1**–**16** are available online.
