# Supplementary material for: Catechol-Type Flavonoids from the Branches of Elaeagnus glabra f. oxyphylla Exert Antioxidant Activity and an Inhibitory Effect on Amyloid-β Aggregation
Source: Molecules. 2020 Oct 23;25(21):4917. doi: 10.3390/molecules25214917 (PMC7660689; doi:10.3390/molecules25214917)
Supplement: Supplementary file 1 [file molecules-25-04917-s001.pdf]

Catechol-type flavonoids from the branches of *Elaeagnus glabra* f. *oxyphylla* exert the inhibitory effect on amyloid-β aggregation and antioxidant activity

Yu Jin Kim [1], Eunjin Sohn [1], Joo-Hwan Kim [2], MinKyun Na [3] and Soo-Jin Jeong [1,*]

[1] Clinical Medicine Division, Korea Institute of Oriental Medicine, Daejeon 34054, Republic of Korea
[2] Department of Life Science, Gachon University, Seongnam, Gyeonggi-do 13120, Republic of Korea
[3] College of Pharmacy, Chungnam National University, Daejeon 34134, Republic of Korea
[*] Correspondence: sjijeong@kiom.re.kr; Tel.: +82-42-868-9651

Received: date; Accepted: date; Published: date

List of Supplementary Materials

Figure S1. ^{1}H-NMR spectrum of compound **1** (DMSO-d_6, 500 MHz)
Figure S2. ^{1}H-NMR spectrum of compound **2** (CD_3OD, 500 MHz)
Figure S3. ^{13}C-NMR spectrum of compound **2** (CD_3OD, 125 MHz)
Figure S4. ^{1}H-NMR spectrum of compound **3** (CD_3OD, 500 MHz)
Figure S5. ^{1}H-NMR spectrum of compound **4** (CD_3OD, 500 MHz)
Figure S6. ^{13}C-NMR spectrum of compound **4** (CD_3OD, 125 MHz)
Figure S7. ^{1}H-NMR spectrum of compound **5** (CD_3OD, 500 MHz)
Figure S8. ^{13}C-NMR spectrum of compound **5** (CD_3OD, 125 MHz)
Figure S9. ^{1}H-NMR spectrum of compound **6** (CD_3OD, 500 MHz)
Figure S10. ^{13}C-NMR spectrum of compound **6** (CD_3OD, 125 MHz)
Figure S11. ^{1}H-NMR spectrum of compound **7** (CD_3OD, 500 MHz)
Figure S12. ^{13}C-NMR spectrum of compound **7** (CD_3OD, 125 MHz)
Figure S13. ^{1}H-NMR spectrum of compound **8** (CD_3OD, 500 MHz)
Figure S14. ^{13}C-NMR spectrum of compound **8** (CD_3OD, 125 MHz)
Figure S15. HMBC spectrum of compound **8** (CD_3OD)
Figure S16. ^{1}H-NMR spectrum of compound **9** (CD_3OD, 500 MHz)
Figure S17. ^{13}C-NMR spectrum of compound **9** (CD_3OD, 125 MHz)
Figure S18. ^{1}H-NMR spectrum of compound **10** (CD_3OD, 500 MHz)
Figure S19. ^{13}C-NMR spectrum of compound **10** (CD_3OD, 125 MHz)
Figure S20. ^{1}H-NMR spectrum of compound **11** (CD_3OD, 500 MHz)
Figure S21. ^{13}C-NMR spectrum of compound **11** (CD_3OD, 125 MHz)
Figure S22. ^{1}H-NMR spectrum of compound **12** (CD_3OD, 500 MHz)
Figure S23. ^{13}C-NMR spectrum of compound **12** (CD_3OD, 125 MHz)
Figure S24. ^{1}H-NMR spectrum of compound **13** (CD_3OD, 500 MHz)
Figure S25. ^{13}C-NMR spectrum of compound **13** (CD_3OD, 125 MHz)
Figure S26. ^{1}H-NMR spectrum of compound **14** (CD_3COCD_3, 500 MHz)
Figure S27. ^{13}C-NMR spectrum of compound **14** (CD_3COCD_3, 125 MHz)
Figure S28. ^{1}H-NMR spectrum of compound **15** (CD_3COCD_3, 500 MHz)
Figure S29. ^{13}C-NMR spectrum of compound **15** (CD_3COCD_3, 125 MHz)
Figure S30. HR ESI-MS spectrum of compound **16**
Figure S31. ^{1}H-NMR spectrum of compound **16** ($CDCl_3$, 500 MHz)
Figure S32. ^{13}C-NMR spectrum of compound **16** ($CDCl_3$, 125 MHz)
Figure S33. HMBC spectrum of compound **16** ($CDCl_3$)

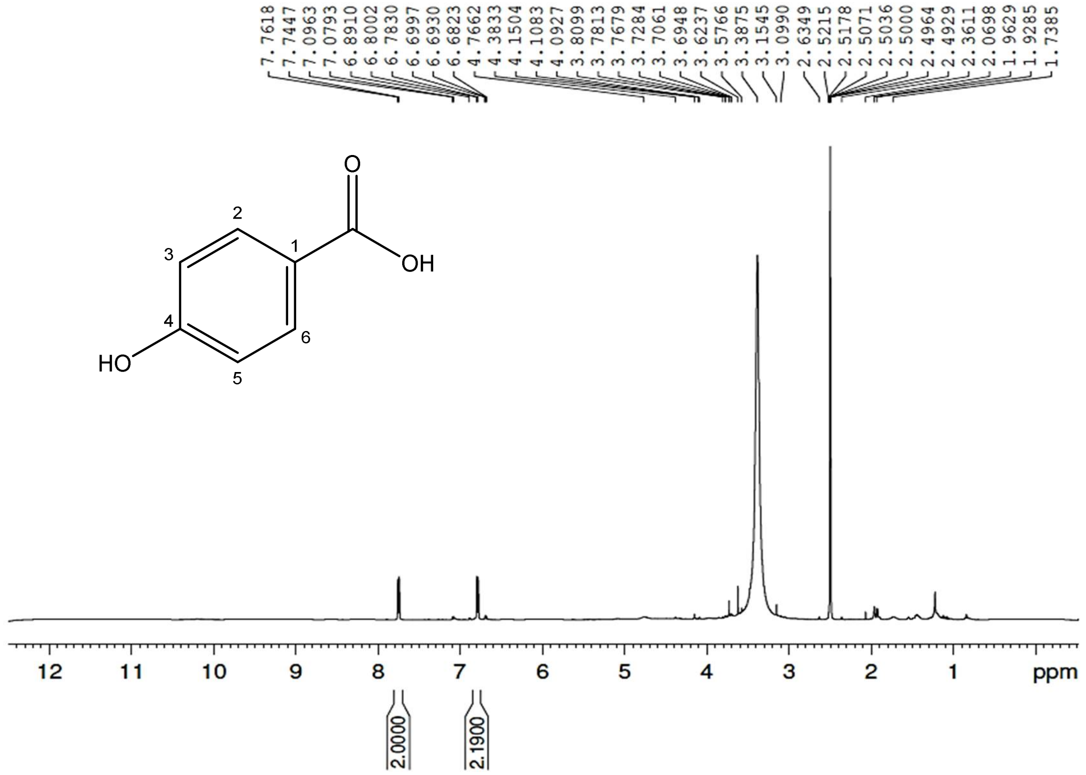

Figure S1. ^{1}H-NMR spectrum of compound 1 (DMSO-d_6, 500 MHz)

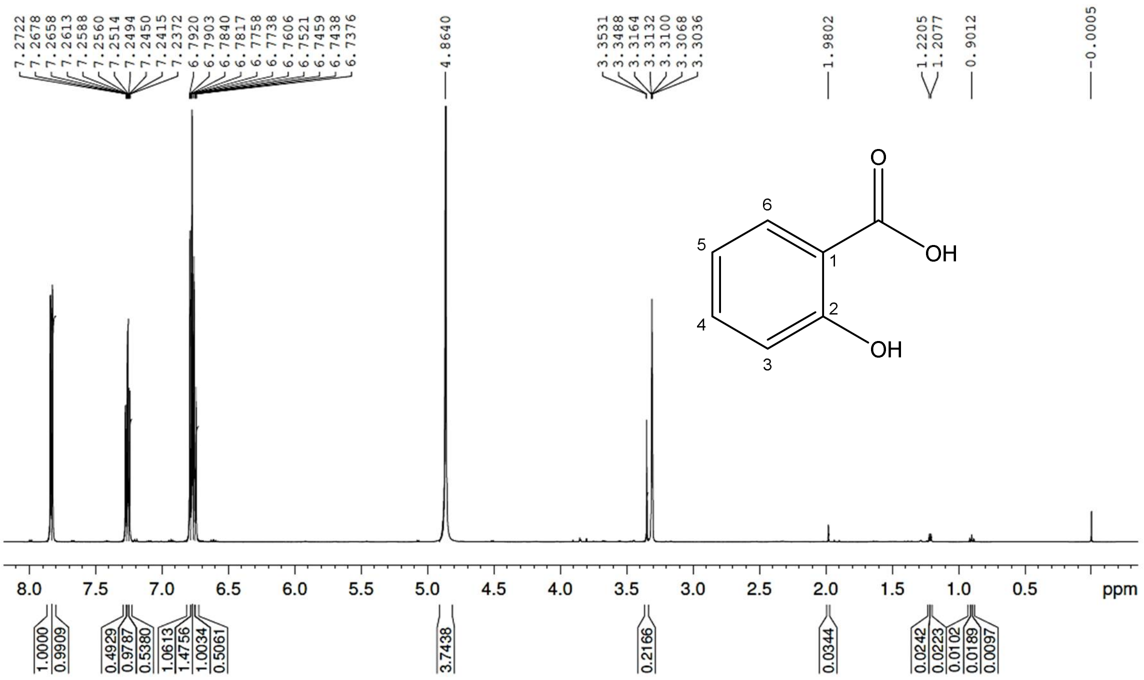

Figure S2. ^{1}H-NMR spectrum of compound 2 (CD_3OD, 500 MHz)

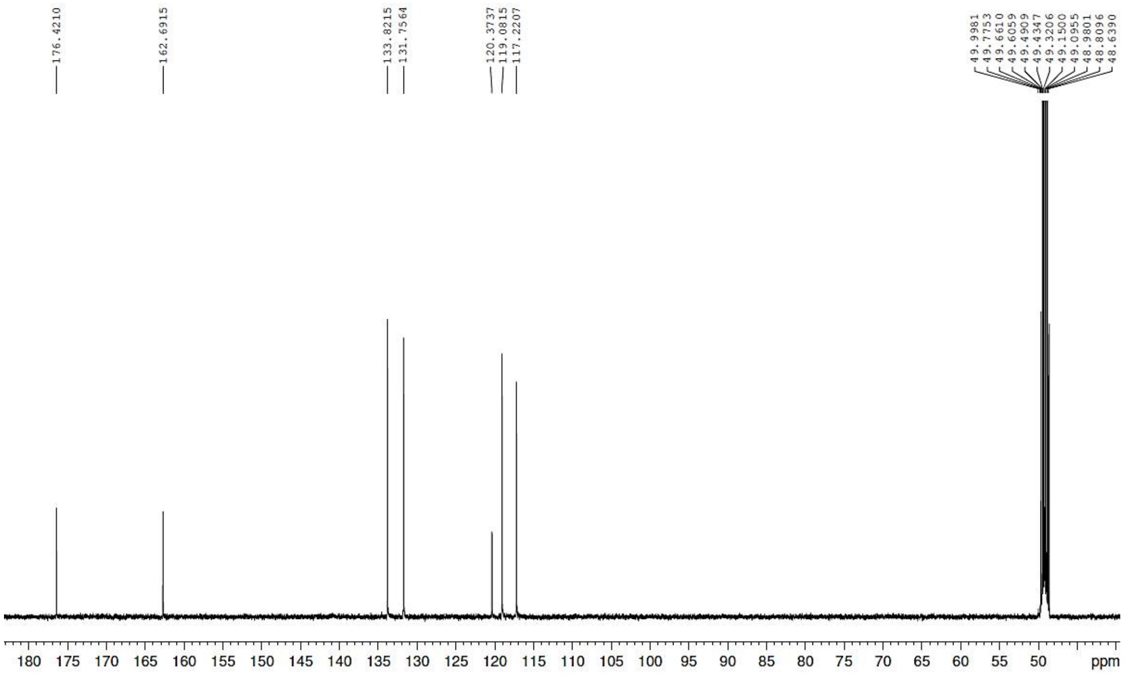

Figure S3. ^{13}**C-NMR spectrum of compound 2 (CD**$_3$**OD, 125 MHz)**

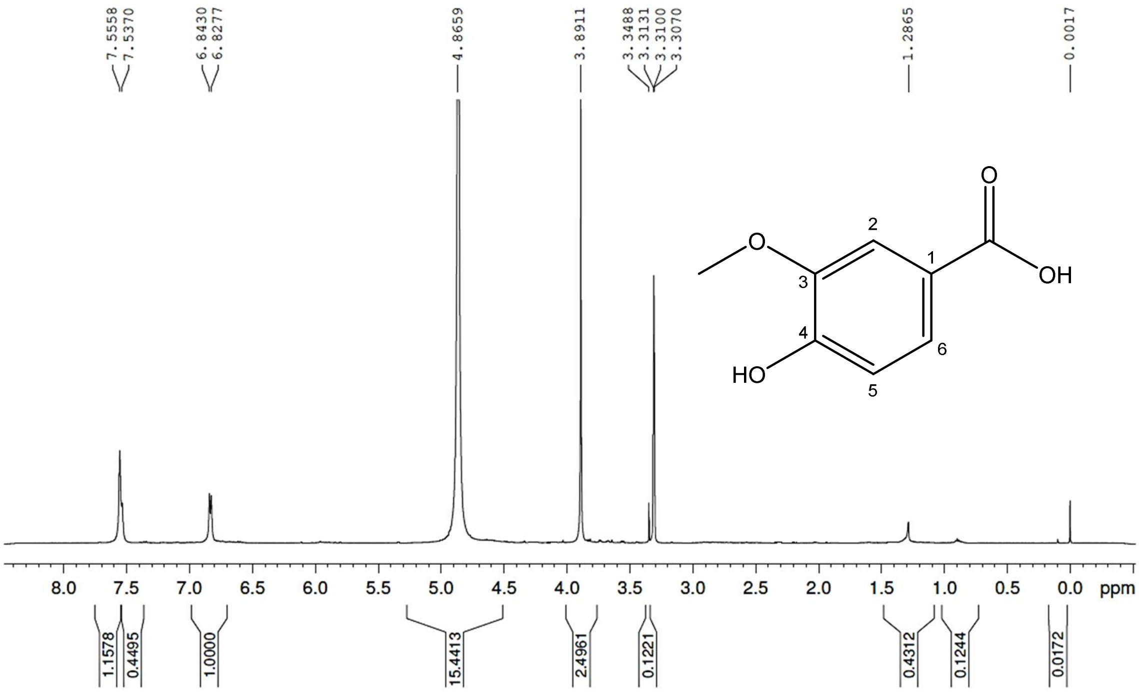

Figure S4. ^{1}**H-NMR spectrum of compound 3 (CD**$_3$**OD, 500 MHz)**

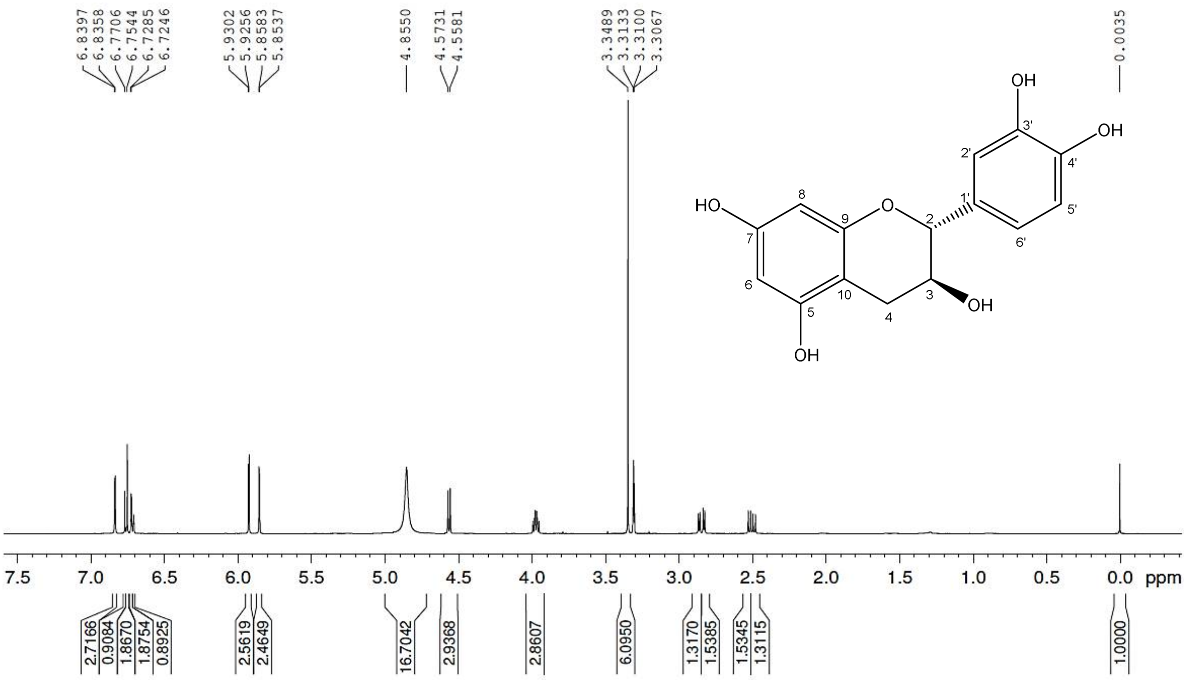

Figure S5. ^1H**-NMR spectrum of compound 4 (**CD_3OD**, 500 MHz)**

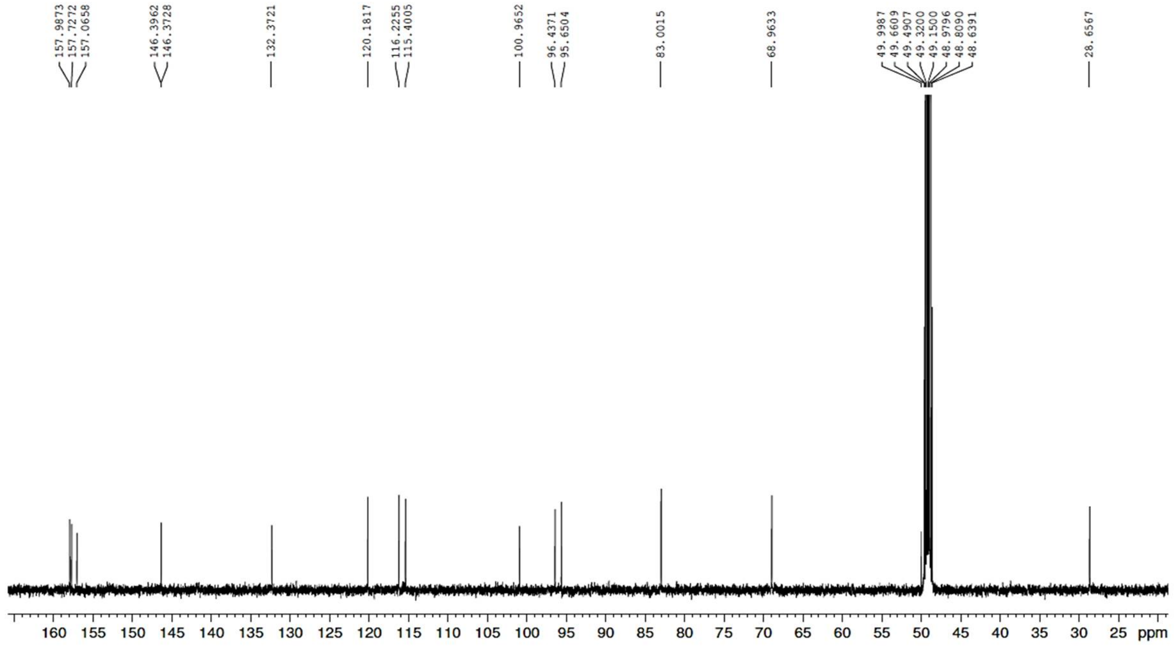

Figure S6. ^{13}C**-NMR spectrum of compound 4 (**CD_3OD**, 125 MHz)**

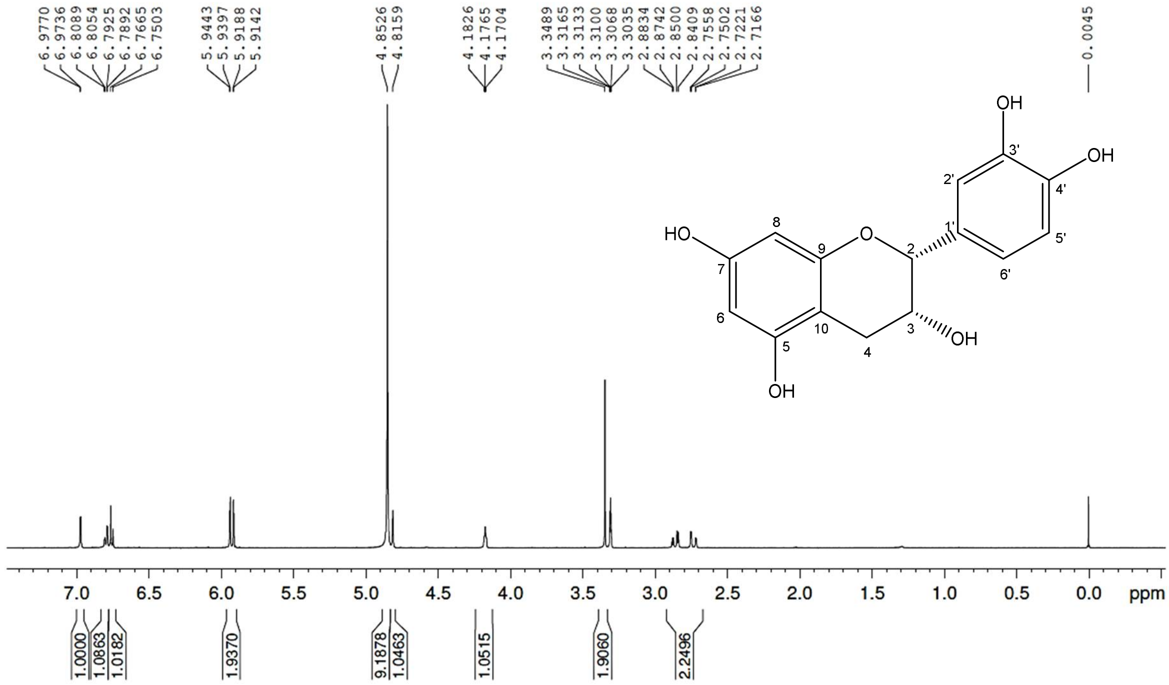

Figure S7. **¹H-NMR spectrum of compound 5 (CD_3OD, 500 MHz)**

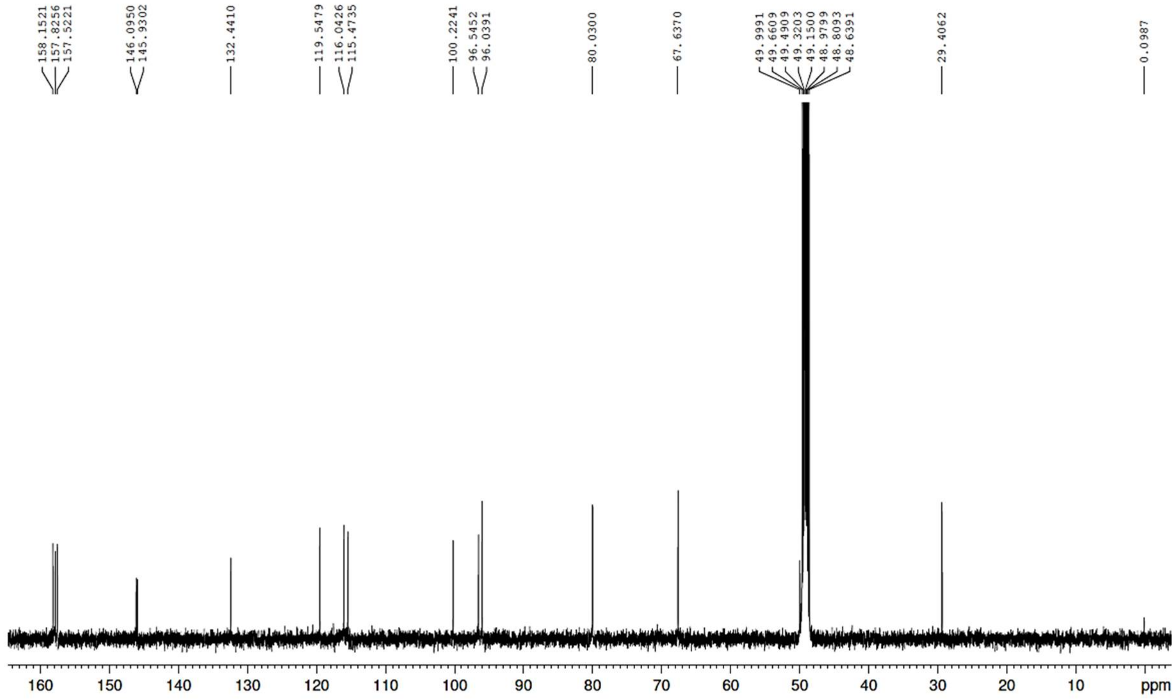

Figure S8. **¹³C-NMR spectrum of compound 5 (CD_3OD, 125 MHz)**

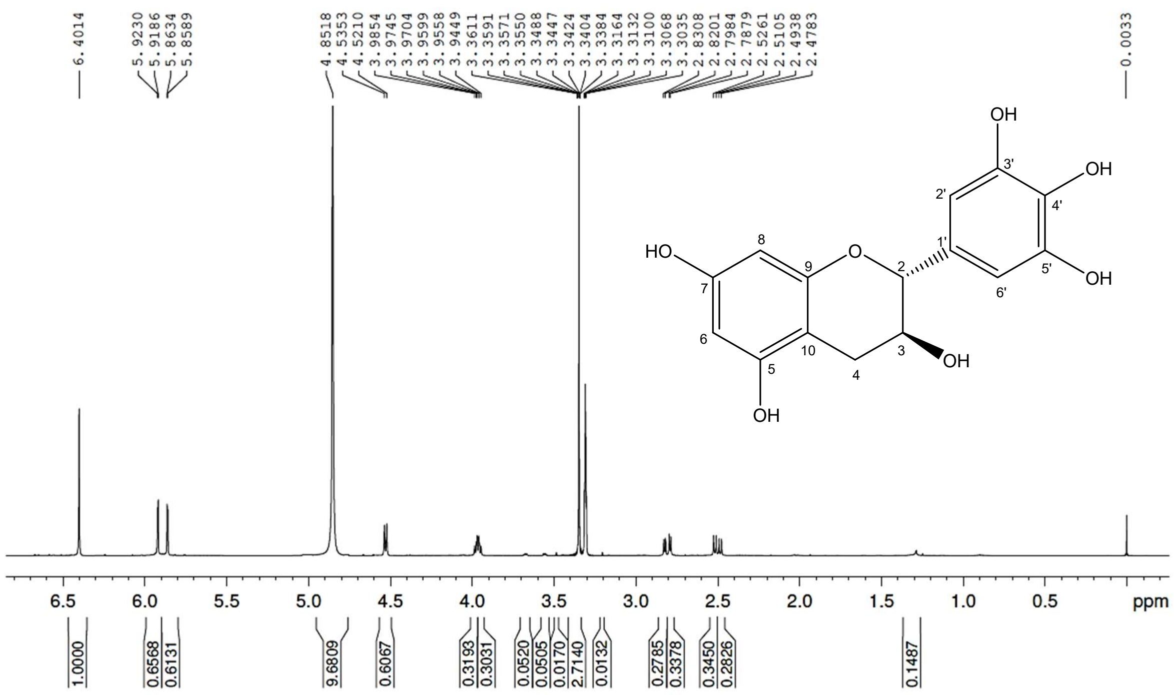

Figure S9. ¹H-NMR spectrum of compound 6 (CD_3OD, 500 MHz)

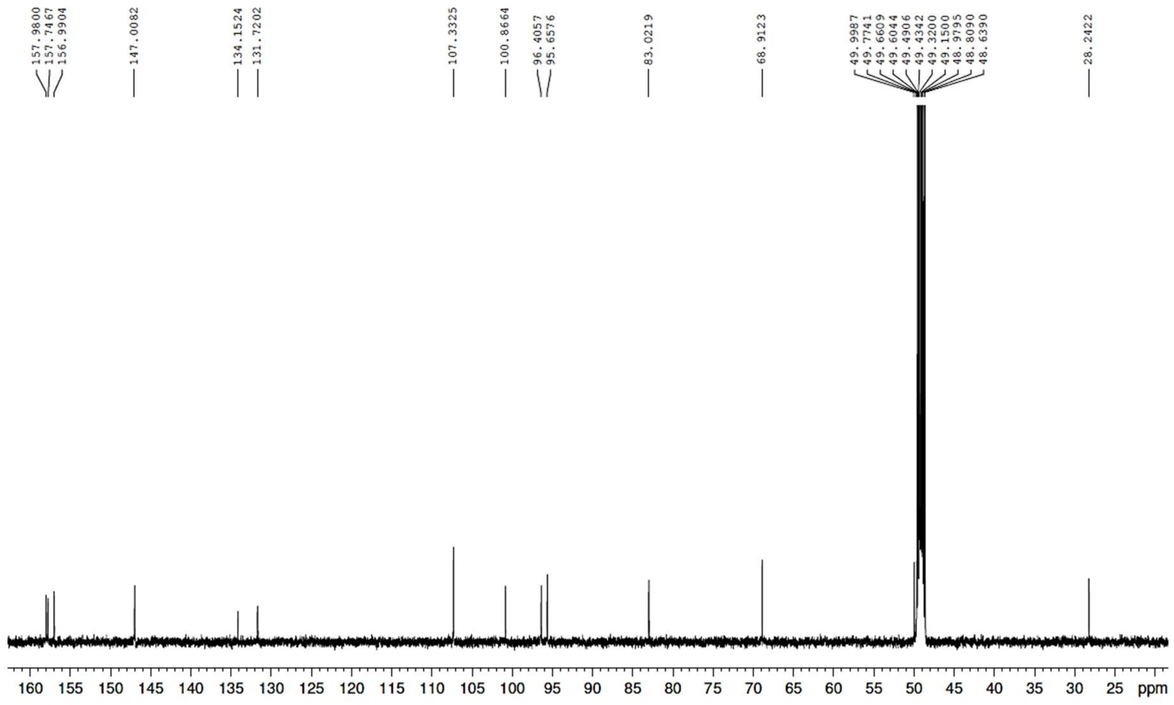

Figure S10. ¹³C-NMR spectrum of compound 6 (CD_3OD, 125 MHz)

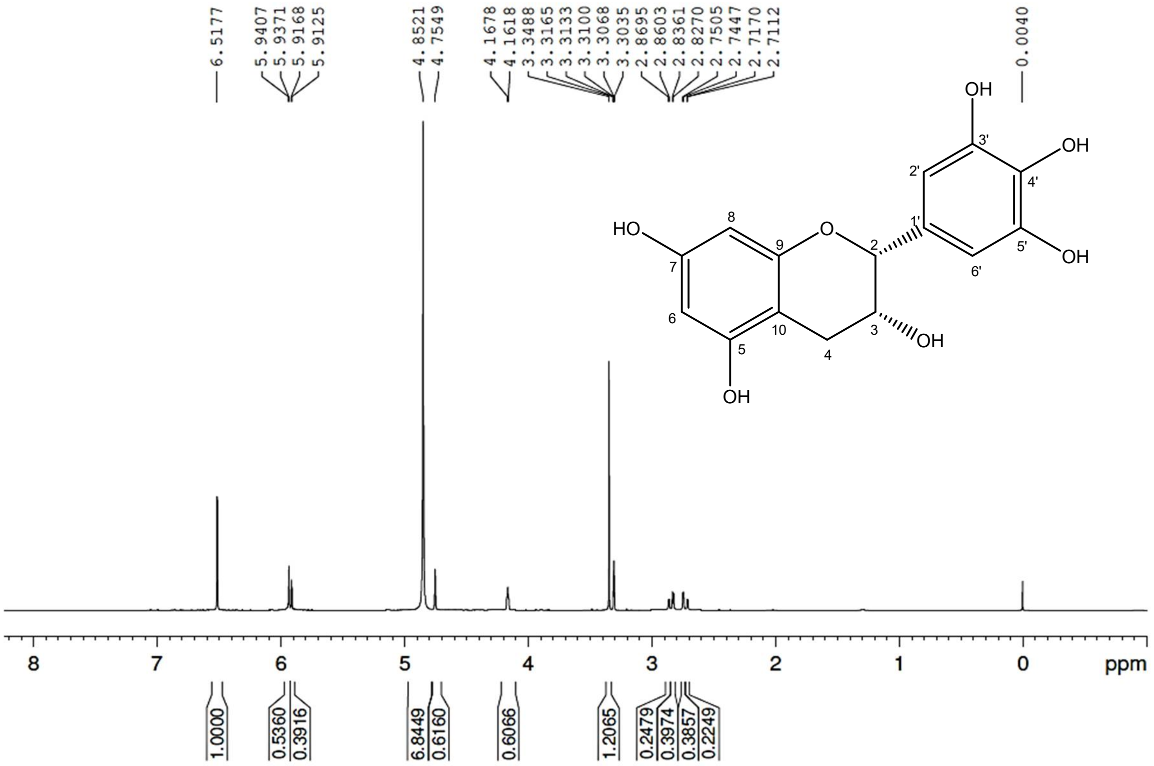

Figure S11. ^{1}H-NMR spectrum of compound 7 (CD_3OD, 500 MHz)

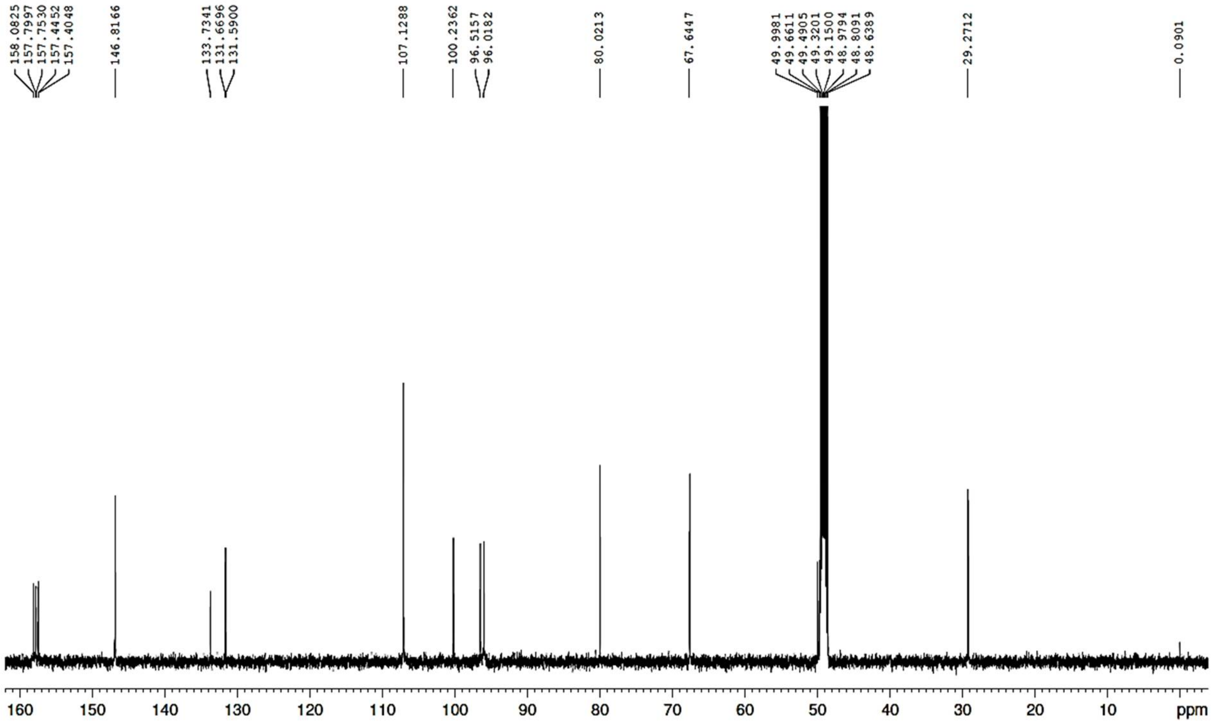

Figure S12. ^{13}C-NMR spectrum of compound 7 (CD_3OD, 125 MHz)

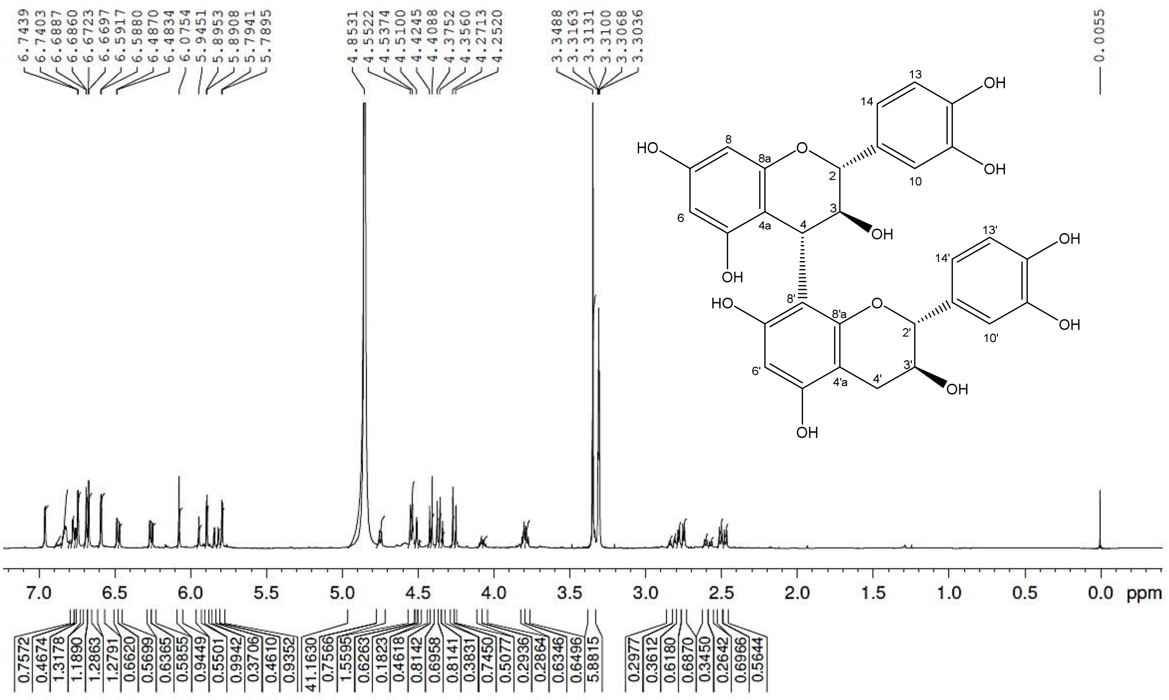

Figure S13. ^{1}H-NMR spectrum of compound 8 (CD_3OD, 500 MHz)

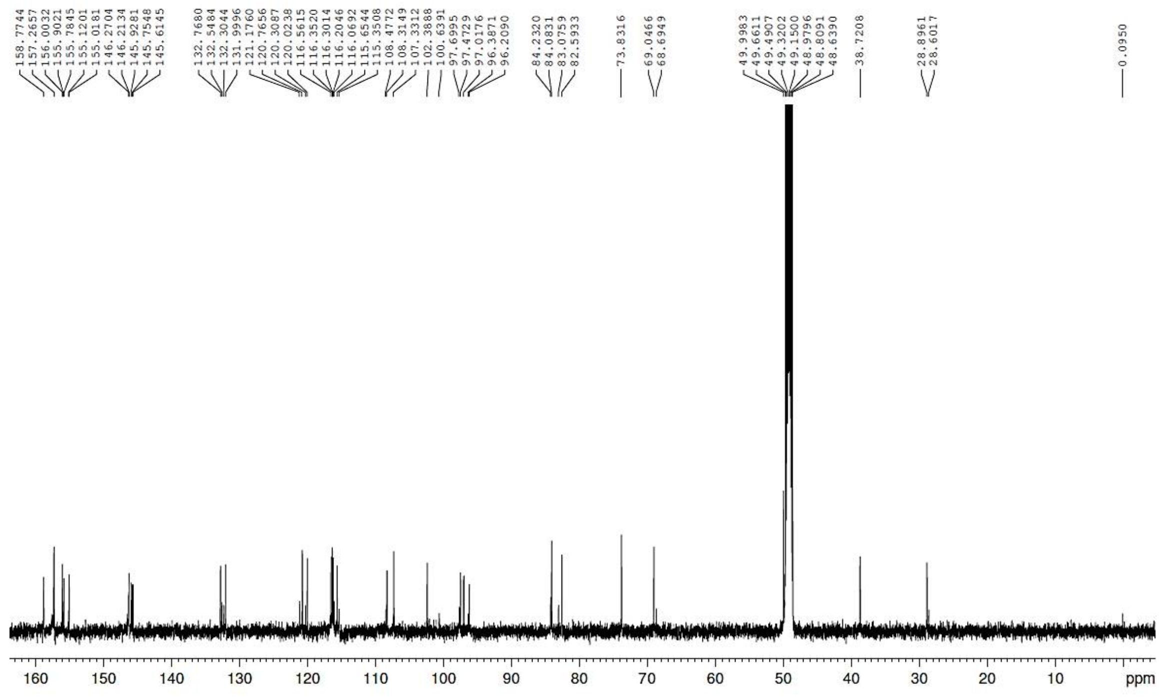

Figure S14. ^{13}C-NMR spectrum of compound 8 (CD_3OD, 125 MHz)

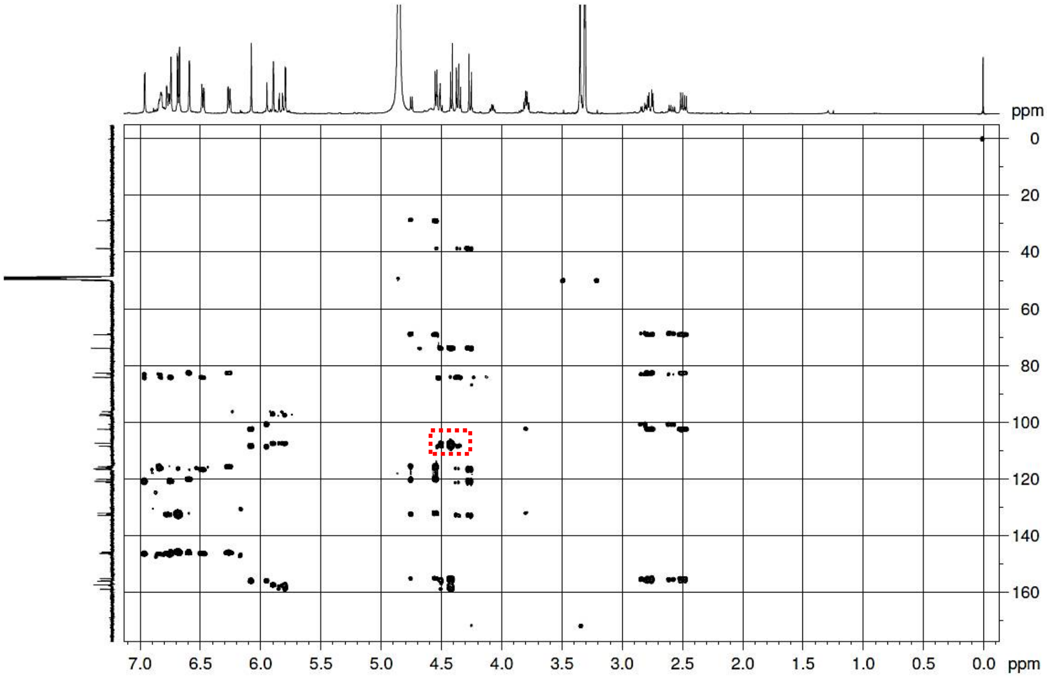

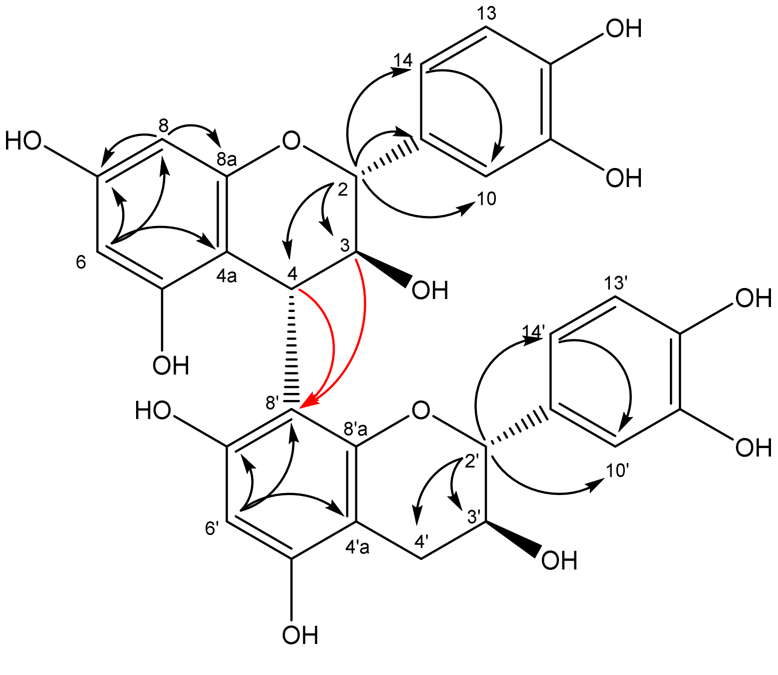

Figure S15. HMBC spectrum of compound 8 (CD_3OD)

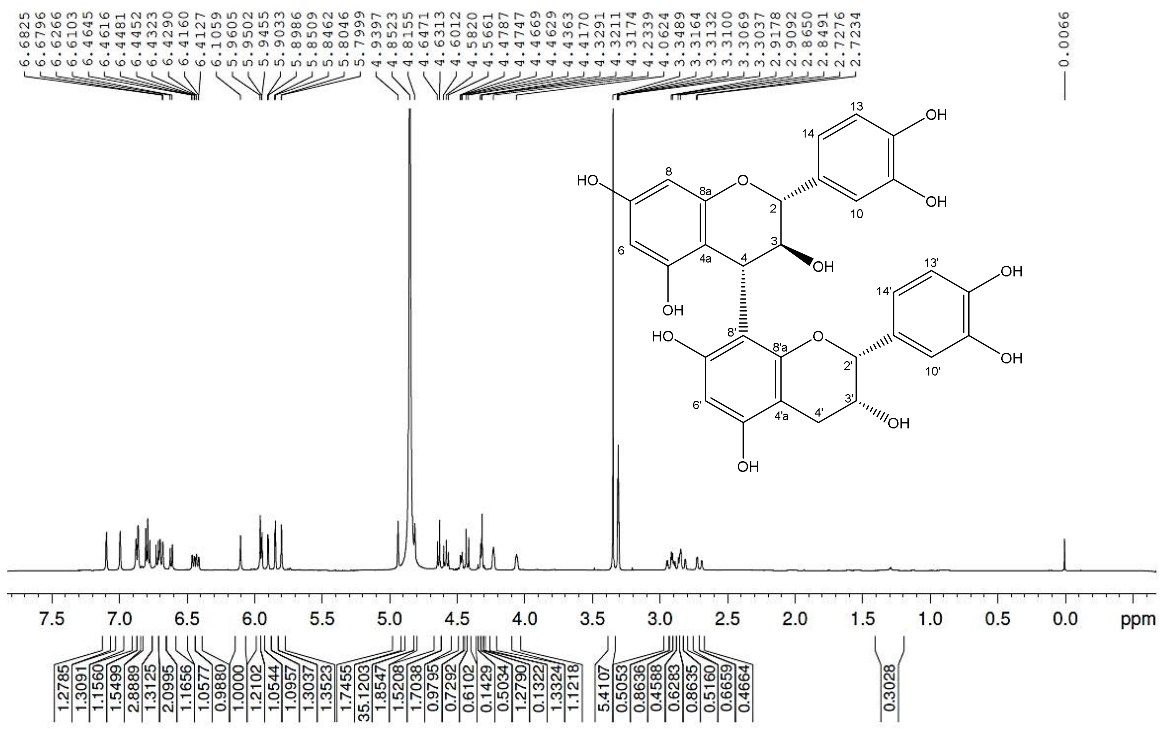

Figure S16. ^{1}H-NMR spectrum of compound 9 (CD_3OD, 500 MHz)

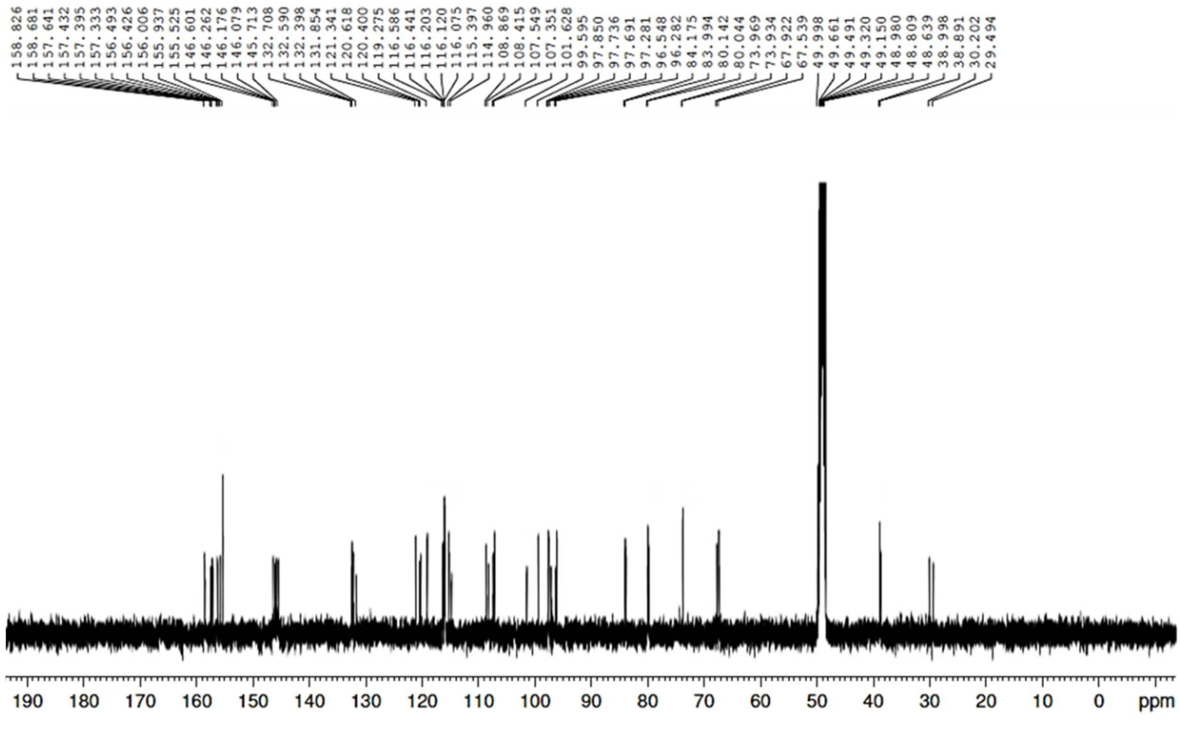

Figure S17. ^{13}C-NMR spectrum of compound 9 (CD_3OD, 125 MHz)

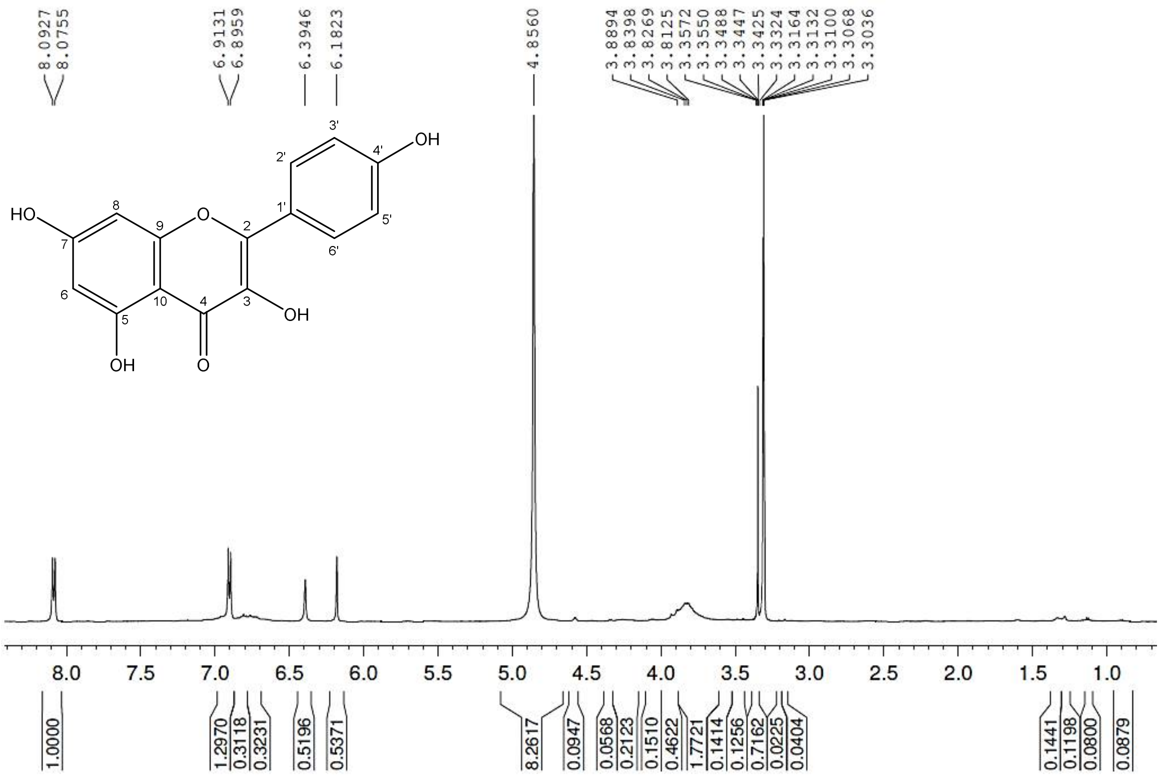

Figure S18. ^{1}H-NMR spectrum of compound 10 (CD_3OD, 500 MHz)

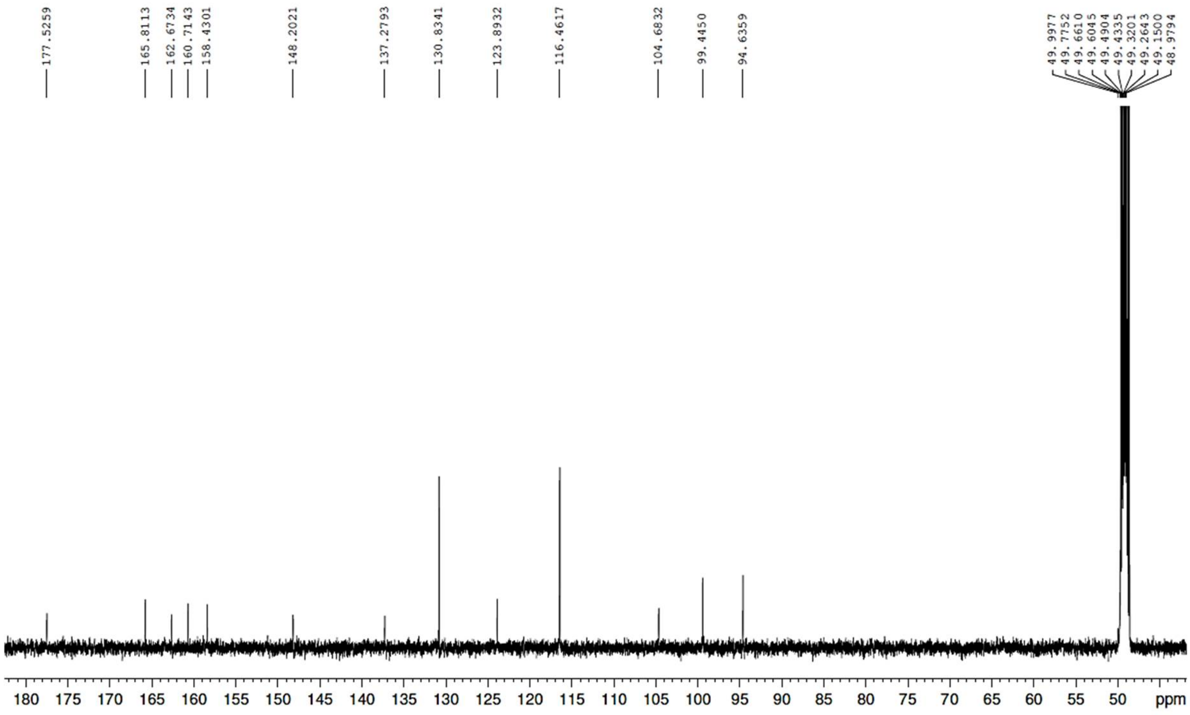

Figure S19. ^{13}C-NMR spectrum of compound 10 (CD_3OD, 125 MHz)

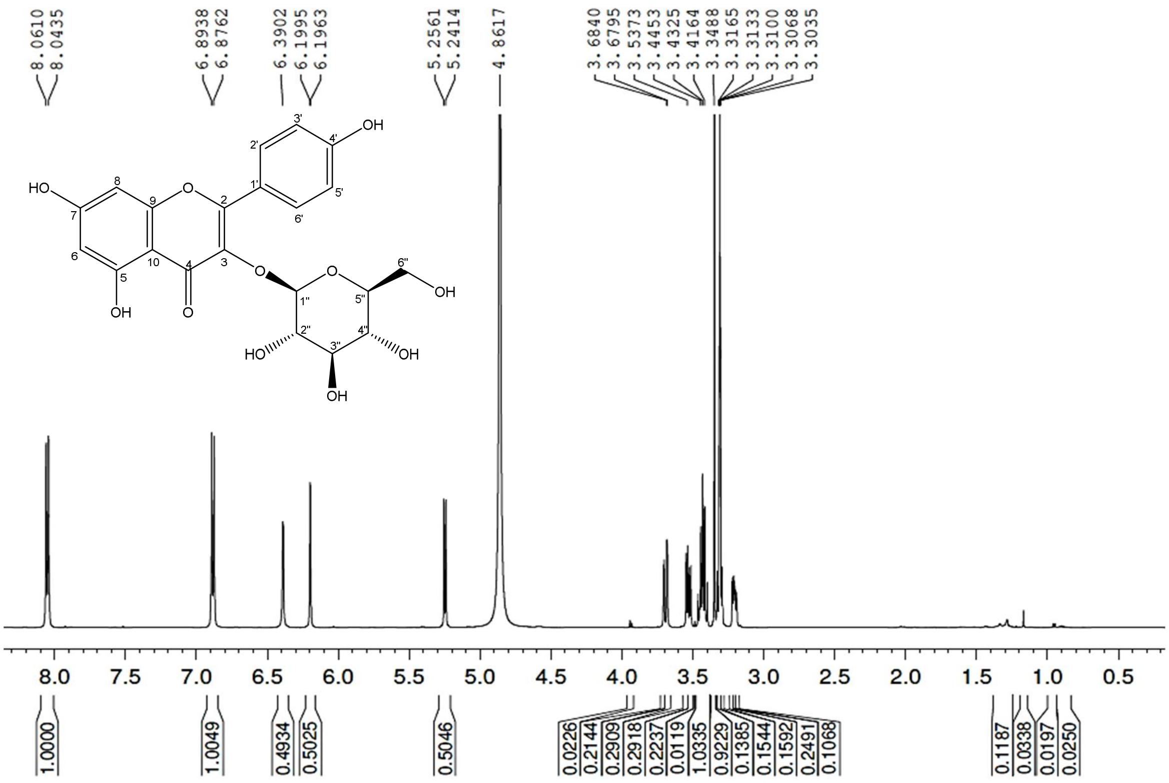

Figure S20. ^{1}H-NMR spectrum of compound 11 (CD_3OD, 500 MHz)

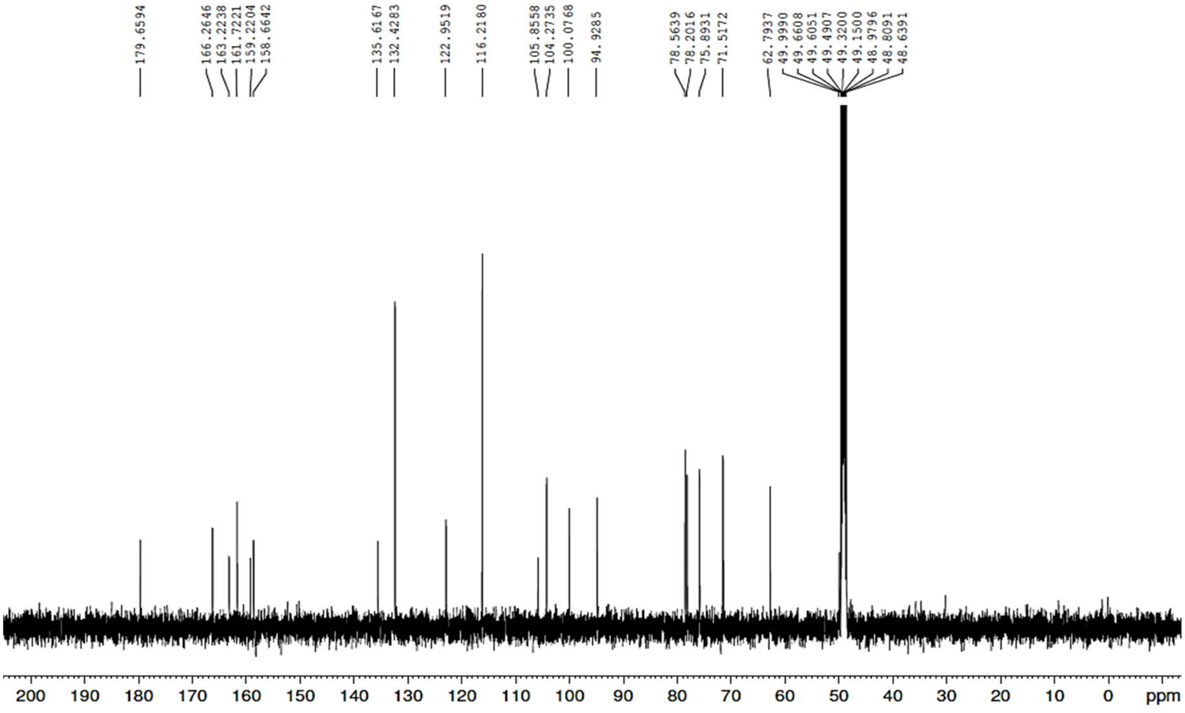

Figure S21. ^{13}C-NMR spectrum of compound 11 (CD_3OD, 125 MHz)

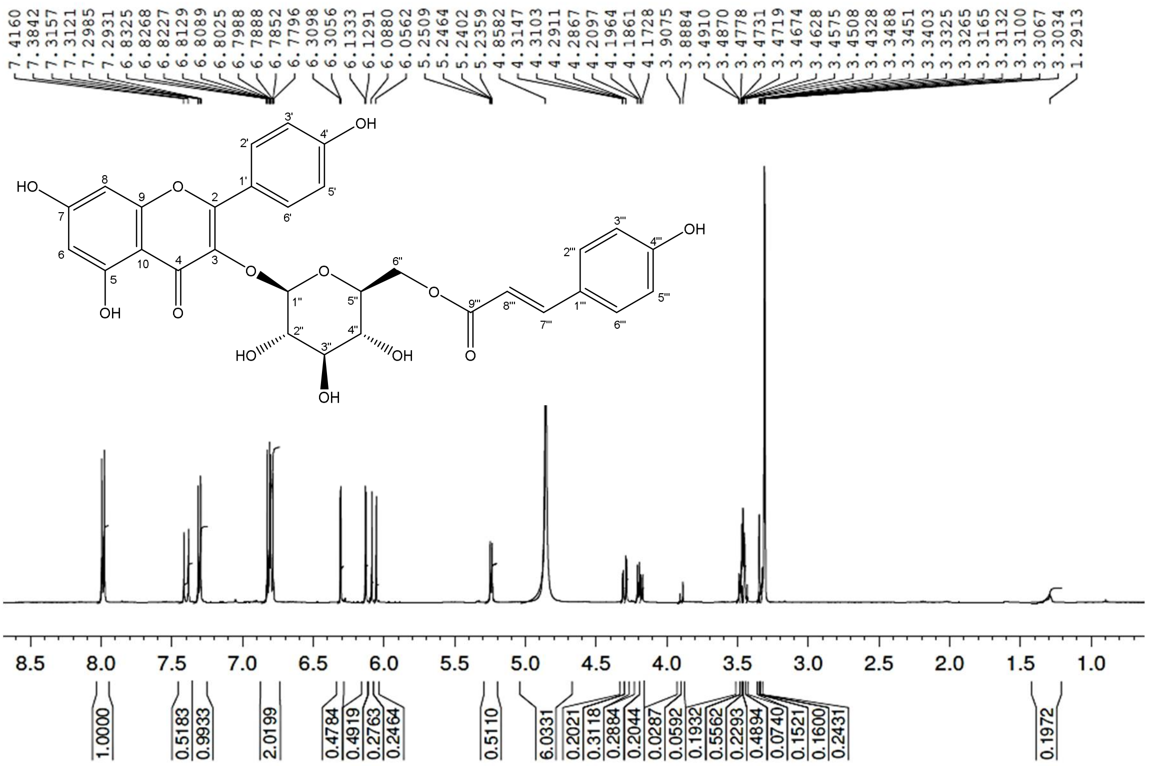

Figure S22. ^{1}H-NMR spectrum of compound 12 (CD_3OD, 500 MHz)

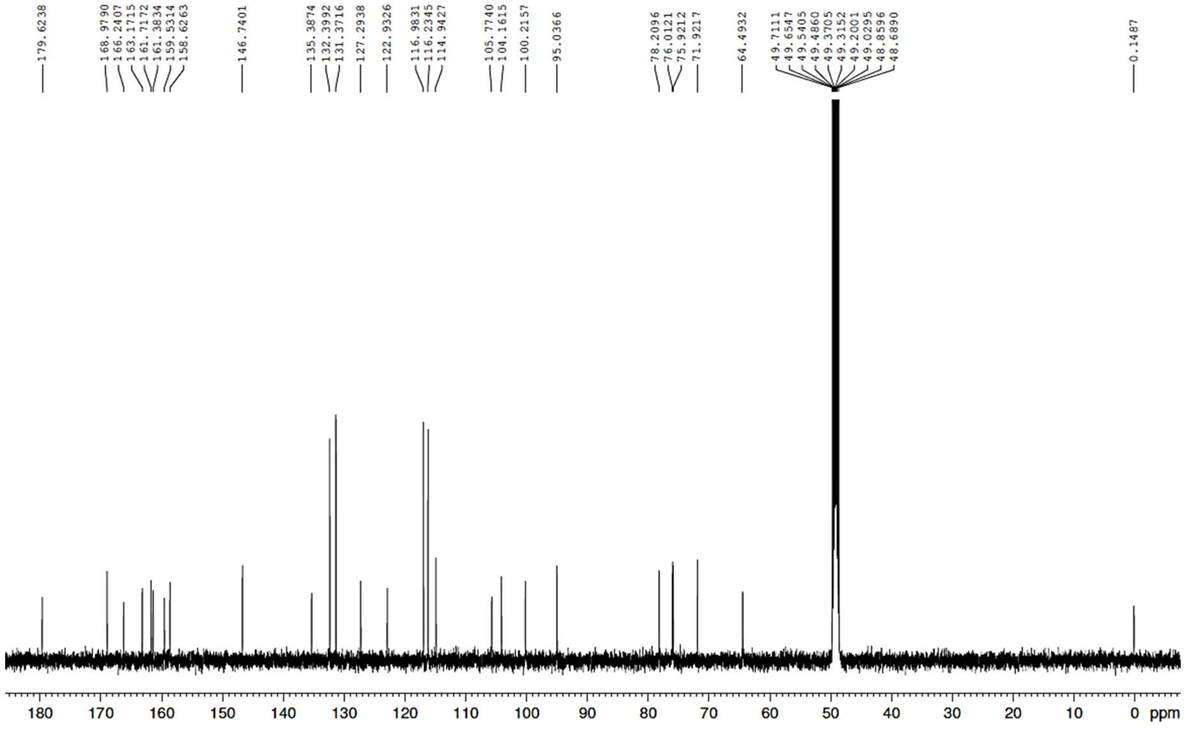

Figure S23. ^{13}C-NMR spectrum of compound 12 (CD_3OD, 125 MHz)

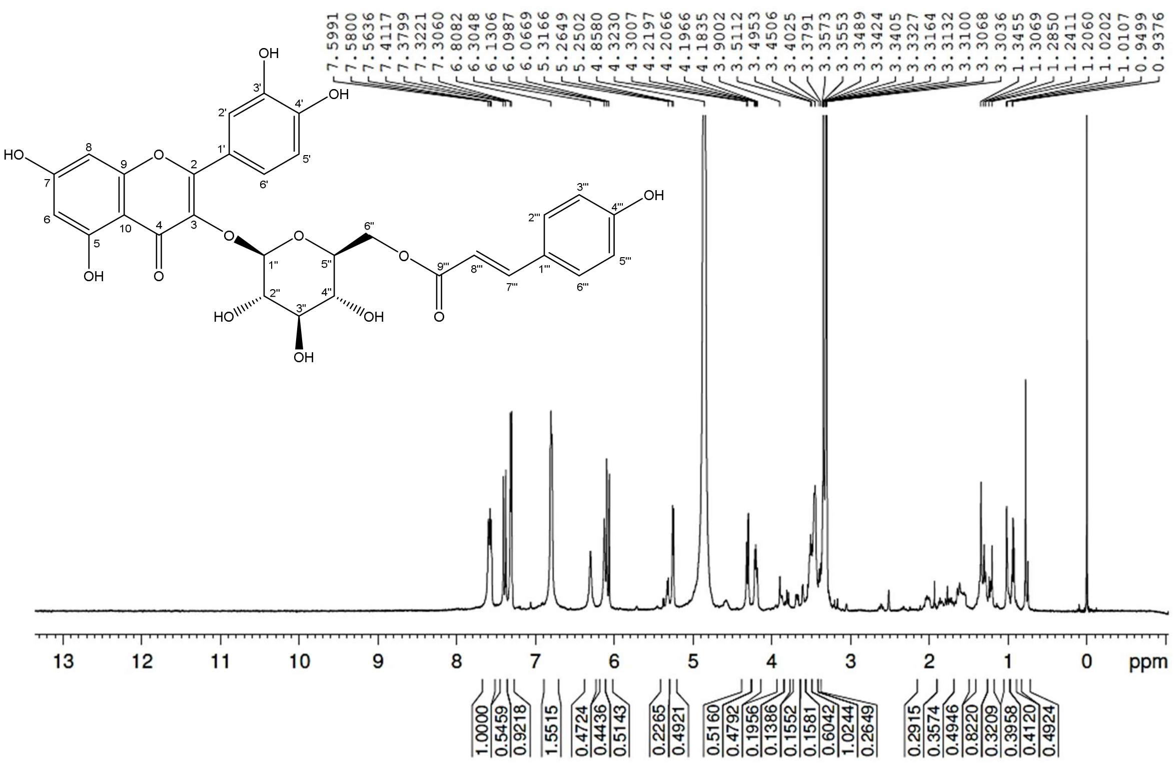

Figure S24. ^{1}H-NMR spectrum of compound 13 (CD_3OD, 500 MHz)

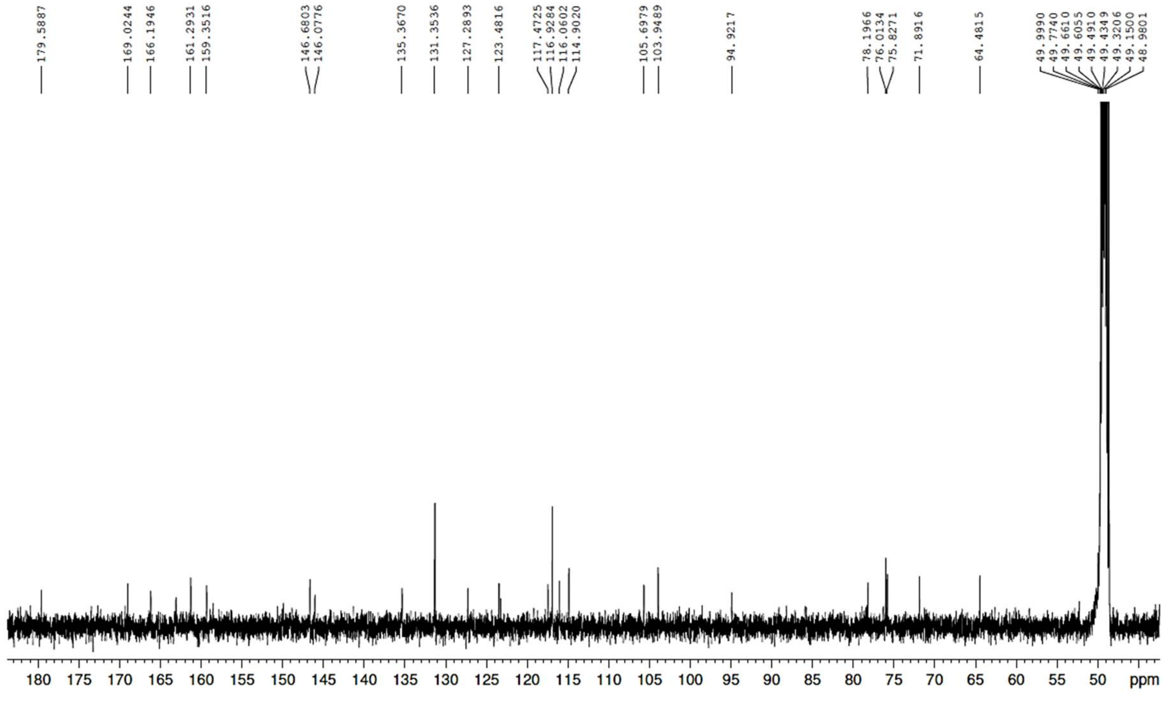

Figure S25. ^{13}C-NMR spectrum of compound 13 (CD_3OD, 125 MHz)

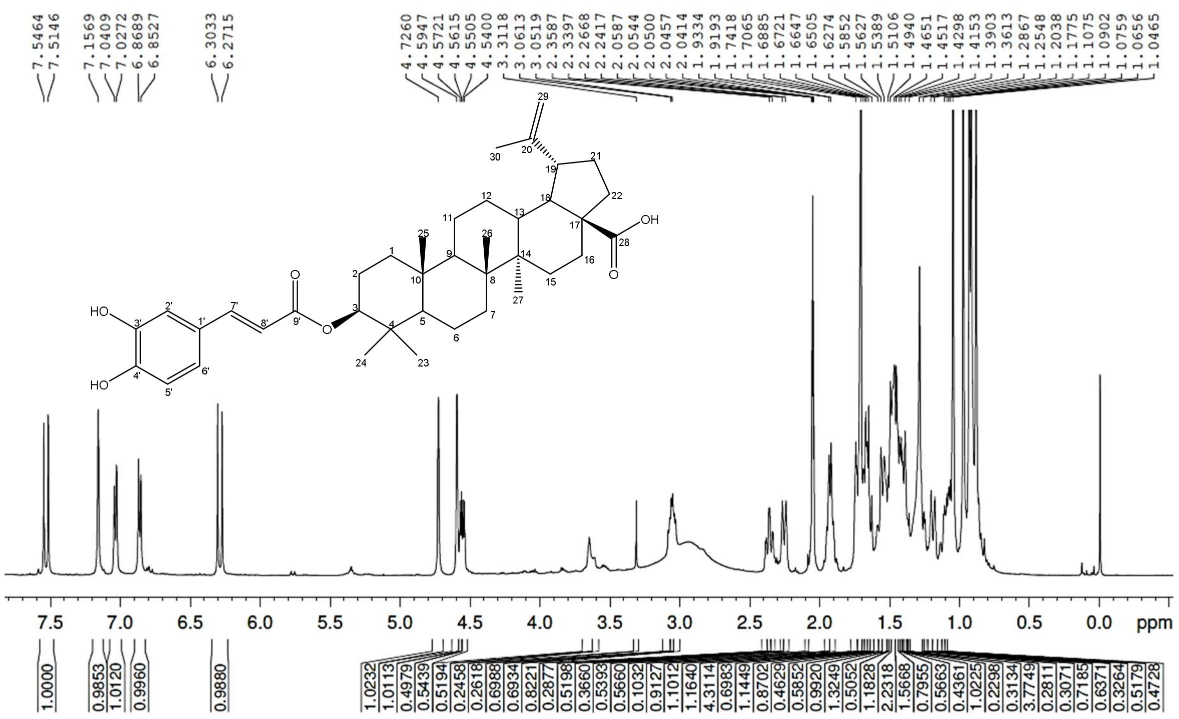

Figure S26. ^{1}H-NMR spectrum of compound 14 (CD_3COCD_3, 500 MHz)

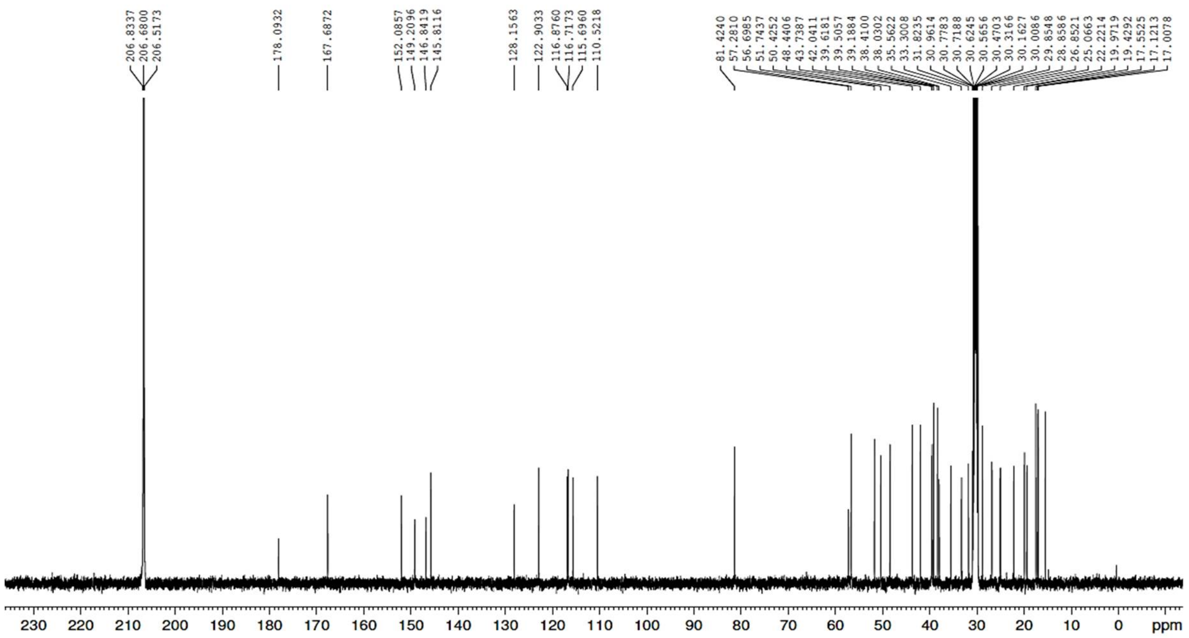

Figure S27. ^{13}C-NMR spectrum of compound 14 (CD_3COCD_3, 125 MHz)

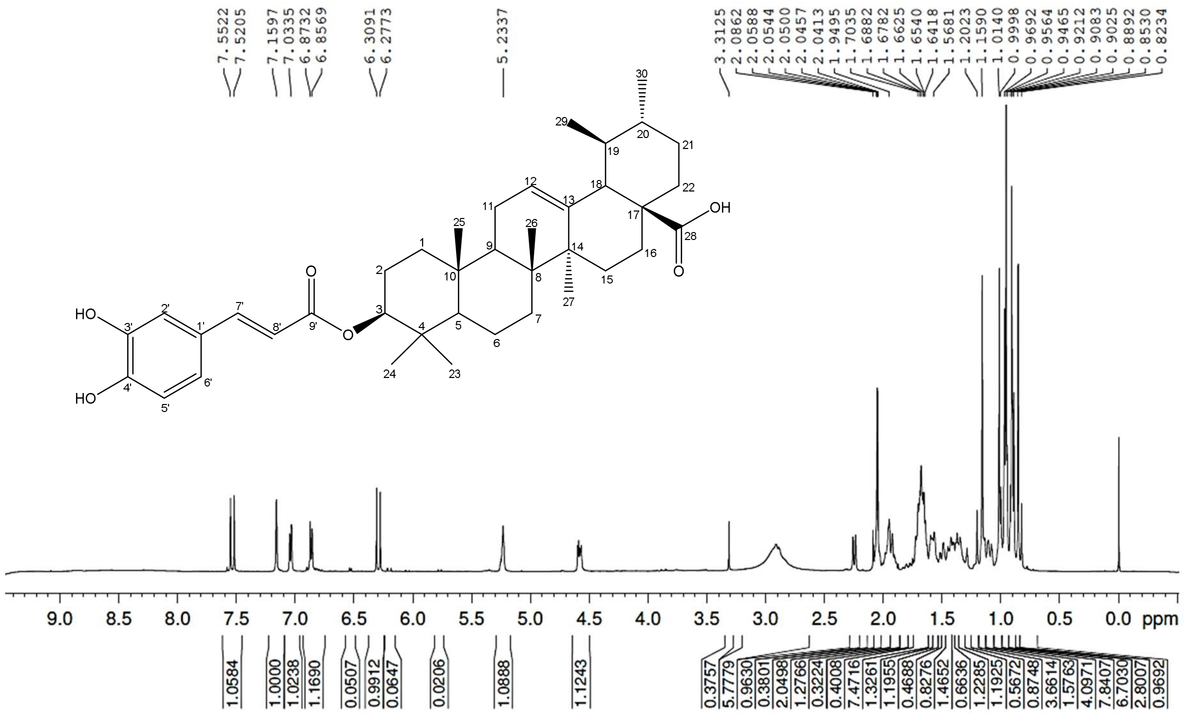

Figure S28. ^{1}H**-NMR spectrum of compound 15 (**CD_3COCD_3**, 500 MHz)**

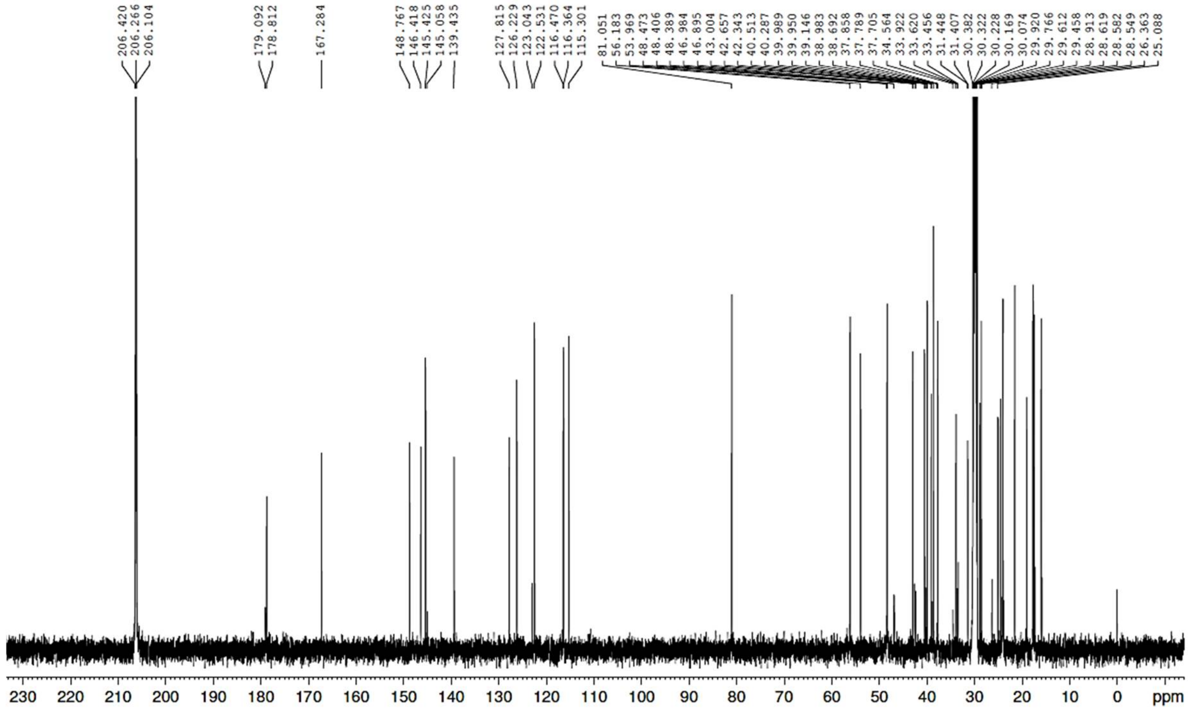

Figure S29. ^{13}C**-NMR spectrum of compound 15 (**CD_3COCD_3**, 125 MHz)**

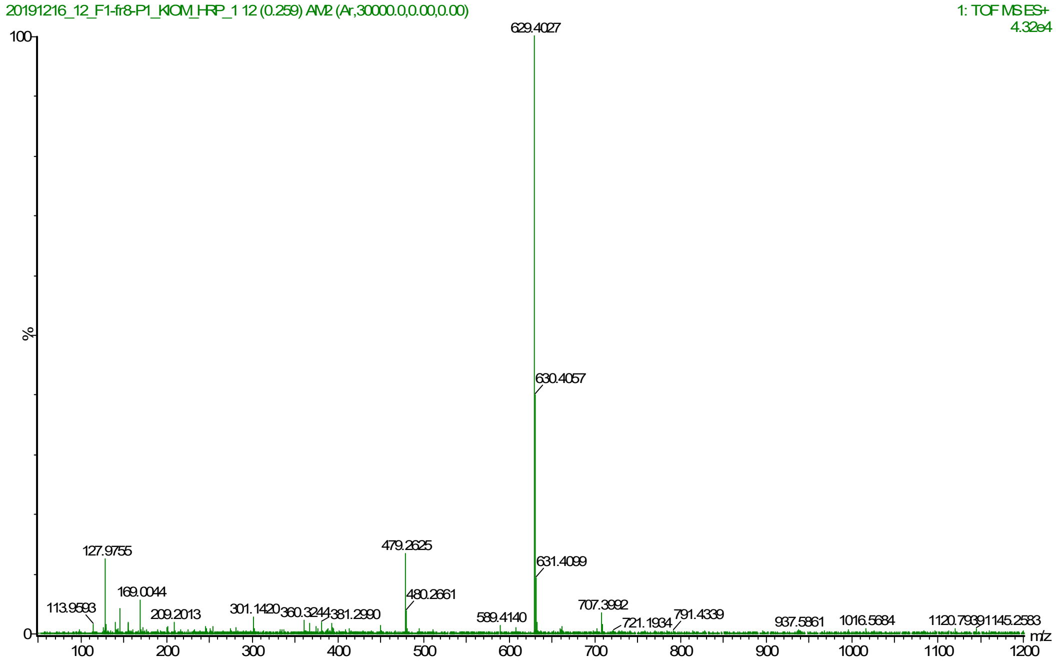

Figure S30. HR ESI-MS spectrum of compound 16

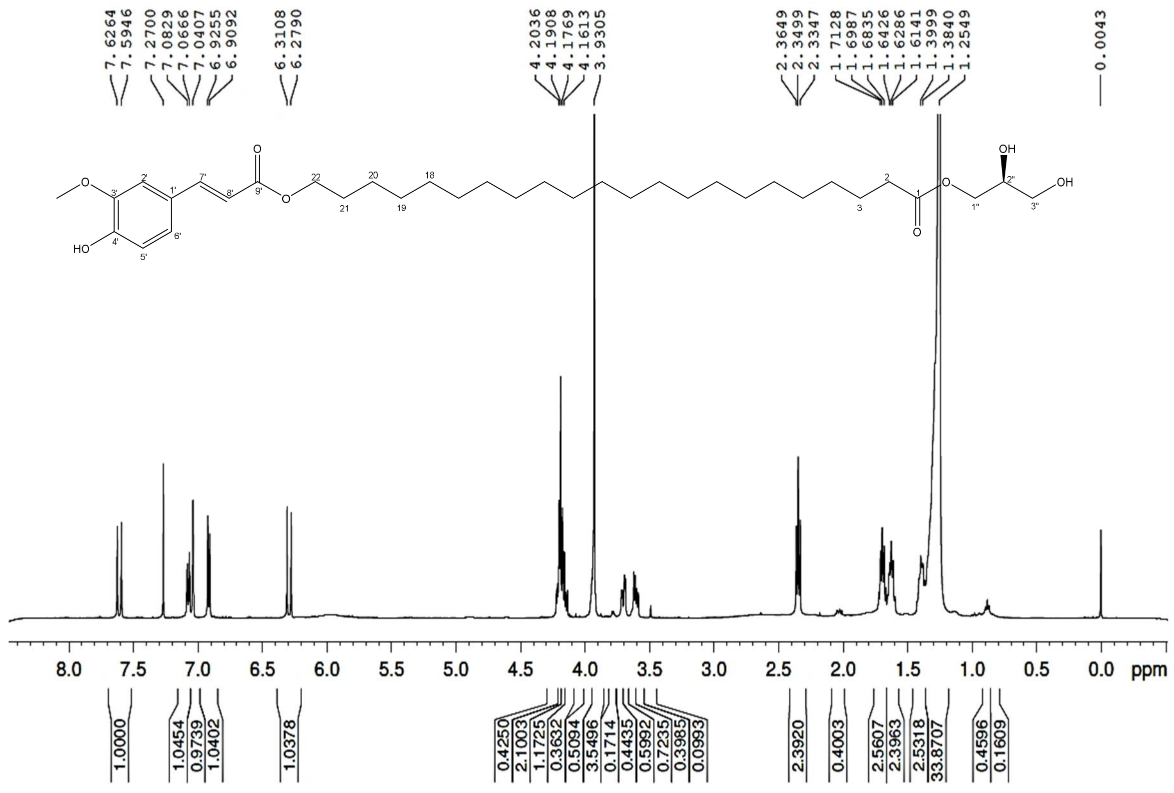

Figure S31. ^{1}**H-NMR spectrum of compound 16 (**$CDCl_3$**, 500 MHz)**

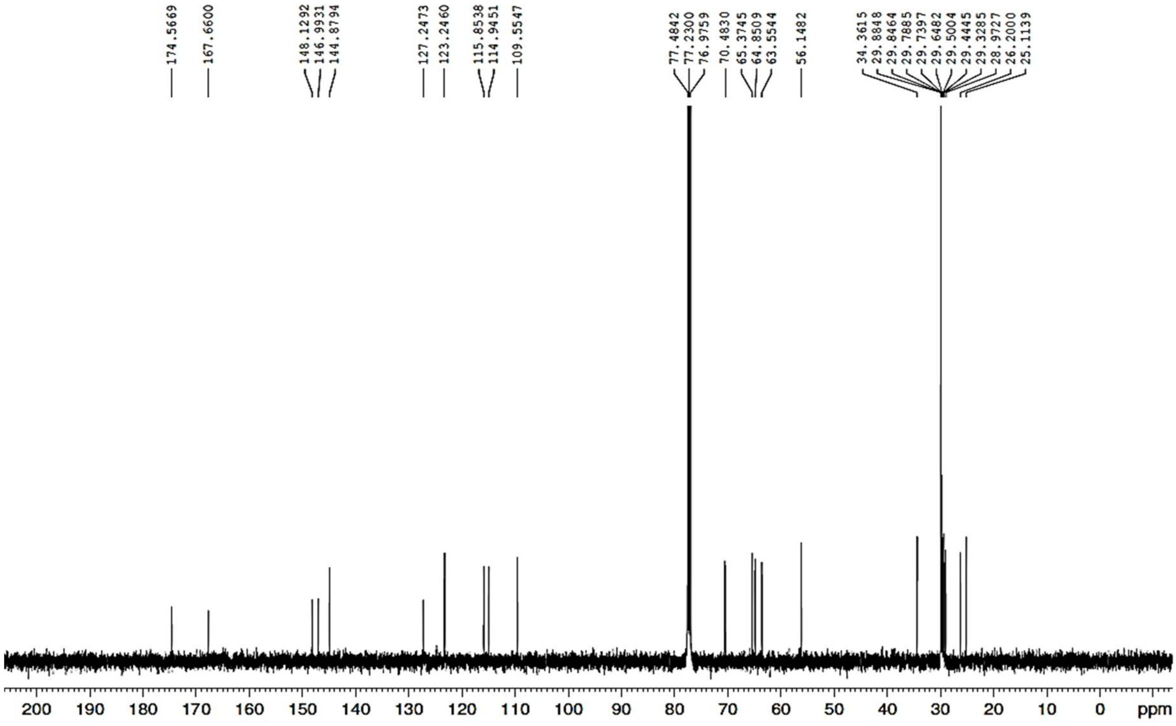

Figure S32. ^{13}**C-NMR spectrum of compound 16 (**$CDCl_3$**, 125 MHz)**

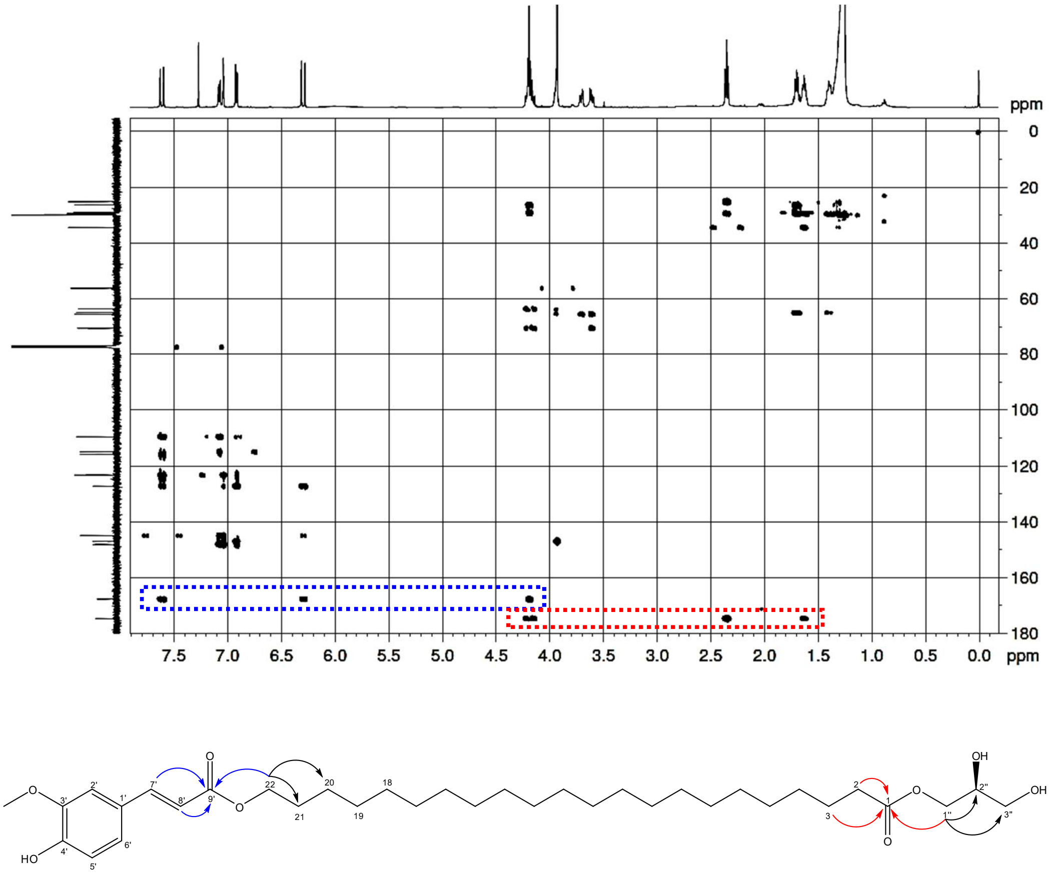

Figure S33. HMBC spectrum of compound 16 ($CDCl_3$)

Physicochemical properties of isolated compounds

4-Hydroxybenzoic acid (1)

White amorphous powder. $C_7H_6O_3$. m.p. 220.4 °C. IR (KBr) ν_{max} cm^{-1}: 3391, 1675, 1595, 1424, 1244. ESI-MS: *m/z* 137.16 [M - H]$^-$. ^{1}H-NMR (DMSO-d_6, 500 MHz): δ 7.75 (2H, d, *J* = 8.6 Hz, H-2,6), 6.79 (2H, d, *J* = 8.6 Hz, H-3,5).

Salicylic acid (2)

White amorphous solid. $C_7H_6O_3$. m.p. 164.5 °C. IR (KBr) ν_{max} cm^{-1}: 3237, 1656, 1612, 759. ESI-MS: *m/z* 137.07 [M - H]$^-$. ^{1}H-NMR (CD_3OD, 500 MHz): δ 7.83 (1H, dd, *J* = 7.8, 1.7 Hz, H-6), 7.25 (1H, td, *J* = 7.8, 1.7 Hz, H-4), 6.78 (1H, dd, *J* = 8.1, 1.1 Hz, H-3), 6.76 (1H, td, *J* = 7.6, 1.1 Hz H-5). ^{13}C-NMR (CD_3OD, 125 MHz): δ 176.4 (C-7), 162.6 (C-2), 133.8 (C-4), 131.7 (C-6), 120.3 (C-1), 119.0 (C-5), 117.2 (C-3).

Vanillic acid (3)

White amorphous powder. $C_8H_8O_4$. m.p. 213 °C. IR (KBr) ν_{max} cm^{-1}: 3484, 2954, 1682, 1434, 1112. HR ESI-MS: *m/z* 191.0300 [M + Na]$^+$ (calcd. for 191. 0320). ^{1}H-NMR (CD_3OD, 500 MHz): δ 7.55 (1H, s, H-2), 7.54 (1H, d, *J* = 7.7 Hz, H-6), 6.83 (1H, d, *J* = 7.7 Hz, H-5), 3.89 (3H, s, OCH_3).

(+)-Catechin (4)

Yellow amorphous powder. $C_{15}H_{14}O_6$. m.p. 174 °C. IR (KBr) ν_{max} cm^{-1}: 3368, 2915, 1628, 1608, 1519, 1282, 1187, 1141, 1100. HR ESI-MS: *m/z* 313.0688 [M + Na]$^+$ (calcd. for 313.0688). $[\alpha]_D^{20}$ +50.7 (*c*=0.4, Me_2CO). ^{1}H-NMR (CD_3OD, 500 MHz): δ 6.83 (1H, d, *J* = 2.0 Hz, H-2′), 6.76 (1H, d, *J* = 8.1 Hz, H-5′), 6.72 (1H, dd, *J* = 8.1, 2.0 Hz, H-6′), 5.93 (1H, d, *J* = 2.3 Hz, H-8), 5.85 (1H, d, *J* = 2.3 Hz, H-6), 4.56 (1H, d, *J* = 7.5 Hz, H-2), 3.98 (1H, ddd, *J* = 5.4, 7.8, 7.8 Hz, H-3), 2.84 (1H, dd, *J* = 16.1, 5.4 Hz, H-4α), 2.51 (1H, dd, *J* = 16.1, 8.2 Hz, H-4β). ^{13}C-NMR (CD_3OD, 125 MHz): δ 157.9 (C-7), 157.7 (C-5), 157.0 (C-9), 146.3 (C-3′, 4′), 132.3 (C-1′), 120.1 (C-6′), 116.2 (C-5′), 115.4 (C-2′), 100.9 (C-10), 96.4 (C-8), 95.6 (C-6), 83.0 (C-2), 68.9 (C-3), 28.6 (C-4).

(–)-Epicatechin (5)

Yellow amorphous powder. $C_{15}H_{14}O_6$. m.p. 234 °C. IR (KBr) ν_{max} cm^{-1}: 3455, 2931, 1625, 1520, 1286, 1184, 1143, 1111. HR ESI-MS: *m/z* 313.0692 [M + Na]$^+$ (calcd. for 313.0688). $[\alpha]_D^{20}$ –18.8 (*c*=0.5, Me_2CO). ^{1}H-NMR (CD_3OD, 500 MHz): δ 6.97 (1H, d, *J* = 1.7 Hz, H-2′), 6.80 (1H, dd, *J* = 8.1, 1.7 Hz, H-6′), 6.76 (1H, d, *J* = 8.1 Hz, H-5′), 5.94 (1H, d, *J* = 2.3 Hz, H-8), 5.91 (1H, d, *J* = 2.3 Hz, H-6), 4.81 (1H, brs, H-2), 4.17 (1H, m, H-3), 2.86 (1H, dd, *J* = 16.8, 4.6 Hz, H-4α), 2.74 (1H, dd, *J* = 16.8, 2.8 Hz, H-4β). ^{13}C-NMR (CD_3OD, 125 MHz): δ 158.1 (C-7), 157.8 (C-5), 157.5 (C-9), 146.0 (C-3′), 145.9 (C-4′), 132.4 (C-1′), 119.5 (C-6′), 116.0 (C-5′), 115.4 (C-2′), 100.2 (C-10), 96.5 (C-8), 96.0 (C-6), 80.0 (C-2), 67.6 (C-3), 29.4 (C-4).

(+)-Gallocatechin (6)

Yellow amorphous powder. $C_{15}H_{14}O_7$. m.p. 197 °C. IR (KBr) ν_{max} cm^{-1}: 3363, 2936, 1625, 1519, 1462, 1344, 1143, 1031. HR ESI-MS: *m/z* 329.0640 [M + Na]$^+$ (calcd. for 329.0637). $[\alpha]_D^{22}$ +12.9 (*c*=0.2, MeOH). ^{1}H-NMR (CD_3OD, 500 MHz): δ 6.40 (2H, s, H-2′,6′), 5.92 (1H, d, *J* = 2.2 Hz, H-

8), 5.86 (1H, d, *J* = 2.2 Hz, H-6), 4.53 (1H, d, *J* = 7.2 Hz, H-2), 3.96 (1H, m, H-3), 2.81 (1H, dd, *J* = 16.1, 5.4 Hz, H-4α), 2.50 (1H, dd, *J* = 16.1, 7.8 Hz, H-4β). ^{13}C-NMR (CD_3OD, 125 MHz): δ 157.9 (C-9), 157.7 (C-7), 156.9 (C-5), 147.0 (C-3′,5′), 134.1 (C-4′), 131.7 (C-1′), 107.3 (C-2′,6′), 100.8 (C-10), 96.4 (C-8), 95.6 (C-6), 83.0 (C-2), 68.9 (C-3), 28.2 (C-4).

(–)-Epigallocatechin (7)

Brown amorphous powder. $C_{15}H_{14}O_7$. m.p. 227 °C. IR (KBr) ν_{max} cm^{-1}: 3398, 2954, 1610, 1518, 1468, 1331, 1279, 1151, 1038. HR ESI-MS: *m/z* 329.0621 [M + Na]$^+$ (calcd. for 329.0637). $[\alpha]_D^{22}$ –26.6 (*c*=0.2, MeOH). ^{1}H-NMR (CD_3OD, 500 MHz): δ 6.51 (2H, s, H-2′,6′), 5.94 (1H, d, *J* = 2.2 Hz, H-6), 5.91 (1H, d, *J* = 2.2 Hz, H-8), 4.75 (1H, brs, H-2), 4.16 (1H, m, H-3), 2.85 (1H, dd, *J* = 16.7, 4.6 Hz, H-4α), 2.73 (1H, dd, *J* = 16.7, 2.9 Hz, H-4β). ^{13}C-NMR (CD_3OD, 125 MHz): δ 158.1 (C-7), 157.7 (C-5), 157.4 (C-9), 146.8 (C-3′,5′), 133.7 (C-4′), 131.6 (C-1′), 107.1 (C-2′,6′), 100.2 (C-10), 96.5 (C-6), 96.0 (C-8), 80.0 (C-2), 67.6 (C-3), 29.2 (C-4).

Procyanidin B3 (8)

Brown amorphous powder. $C_{30}H_{26}O_{12}$. m.p. 210 °C (decomp.). IR (KBr) ν_{max} cm^{-1}: 3390, 1610, 1520, 1451, 1282, 1144, 1063, 820. ESI-MS: *m/z* 577.34 [M - H]$^-$. $[\alpha]_D^{25}$ –164.4 (*c*=0.1, MeOH). ^{1}H-NMR (CD_3OD, 500 MHz, 2:1 mixture of rotational isomer), major isomer: δ 6.74 (1H, d, *J* = 1.8 Hz, H-10), 6.68 (1H, d, *J* = 8.2 Hz, H-13), 6.68 (1H, d, *J* = 8.2 Hz, H-13′), 6.59 (1H, d, *J* = 1.9 Hz, H-10′), 6.48 (1H, dd, *J* = 8.2, 1.8 Hz, H-14), 6.26 (1H, dd, *J* = 8.2, 1.8 Hz, H-14′), 6.07 (1H, s, H-6′), 5.89 (1H, d, *J* = 2.3 Hz, H-6), 5.79 (1H, d, *J* = 2.3 Hz, H-8), 4.54 (1H, d, *J* = 7.4 Hz, H-2′), 4.41 (1H, d, *J* = 7.4 Hz, H-4), 4.35 (1H, t, *J* = 9.6 Hz, H-3), 4.26 (1H, d, *J* = 9.6 Hz, H-2), 3.80 (1H, m, H-3′), 2.77 (1H, dd, *J* = 16.3, 5.5 Hz, H-4′α), 2.49 (1H, dd, *J* = 16.3, 8.0 Hz, H-4′β), minor isomer: δ 6.96 (1H, d, *J* = 1.7 Hz, H-10), 6.96 (1H, d, *J* = 1.7 Hz, H-10′), 6.84 (1H, m, H-14′), 6.82 (1H, m, H-14), 6.77 (1H, d, *J* = 8.1 Hz, H-13′), 6.76 (1H, d, *J* = 8.2 Hz, H-13), 5.95 (1H, brs, H-6′), 5.84 (1H, d, *J* = 2.3 Hz, H-6), 5.81 (1H, d, *J* = 2.3 Hz, H-8), 4.75 (1H, d, *J* = 7.2 Hz, H-2′), 4.52 (1H, d, *J* = 9.6 Hz, H-3), 4.51 (1H, d, *J* = 7.8 Hz, H-4), 4.37 (1H, d, *J* = 9.6 Hz, H-2), 2.82 (1H, dd, *J* = 16.2, 5.4 Hz, H-4′α), 2.58 (1H, dd, *J* = 16.2, 7.7 Hz, H-4′β). ^{13}C-NMR (CD_3OD, 125 MHz), major isomer: δ 158.7 (C-8a), 157.2 (C-5,7), 156.0 (C-7′), 155.7 (C-8′a), 155.0 (C-5′), 146.2 (C-12), 145.9 (C-12′), 145.7 (C-11), 145.6 (C-11′), 132.7 (C-9), 131.9 (C-9′), 120.7 (C-14), 120.0 (C-14′), 116.5 (C-10), 116.3 (C-13), 116.2 (C-13′), 115.6 (C-10′), 108.3 (C-8′), 107.3 (C-4a), 102.3 (C-4′a), 97.4 (C-6), 97.0 (C-8), 96.2 (C-6′), 84.0 (C-2), 82.5 (C-2′), 73.8 (C-3), 69.0 (C-3′), 38.7 (C-4), 28.8 (C-4′), minor isomer: δ 158.7 (C-8a), 157.2 (C-5,7), 155.9 (C-7′), 155.7 (C-8′a), 155.1 (C-5′), 146.2 (C-12), 146.2 (C-12′), 145.9 (C-11), 145.9 (C-11′), 132.5 (C-9), 132.3 (C-9′), 121.1 (C-14), 120.3 (C-14′), 116.3 (C-10), 116.2 (C-13), 116.0 (C-13′), 115.3 (C-10′), 108.4 (C-8′), 107.3 (C-4a), 100.6 (C-4′a), 97.7 (C-8), 97.6 (C-6′), 96.3 (C-6), 84.2 (C-2), 83.0 (C-2′), 73.8 (C-3), 68.6 (C-3′), 38.7 (C-4), 28.6 (C-4′).

Procyanidin B4 (9)

Brown amorphous powder. $C_{30}H_{26}O_{12}$. m.p. 210 °C (decomp.). IR (KBr) ν_{max} cm^{-1}: 3373, 1609, 1520, 1450, 1357, 1283, 1144, 1064, 821. ESI-MS: *m/z* 577.37 [M - H]$^-$. $[\alpha]_D^{25}$ –159.4 (*c*=0.1, MeOH). ^{1}H-NMR (CD_3OD, 500 MHz, 1:1.3 mixture of rotational isomer): δ [7.09 (1H, d, *J* = 1.6 Hz), 6.68 (1H, d, *J* = 1.5 Hz), H-10′], [6.99 (1H, d, *J* = 1.8 Hz), 6.70 (1H, d, *J* = 1.7 Hz), H-10], [6.87 (1H, dd, *J* = 8.2, 1.5 Hz), 6.45 (1H, dd, *J* = 8.2, 1.5 Hz), H-14′], [6.87 (1H, dd, *J* = 8.2, 1.5 Hz), 6.42 (1H, dd, *J* = 8.2, 1.7 Hz), H-14], [6.79 (1H, d, *J* = 8.2 Hz), 6.72 (1H, d, *J* = 8.2 Hz), H-13′], [6.78 (1H, d, *J* = 8.2 Hz), 6.62 (1H, d, *J* = 8.2 Hz), H-13], [6.10 (1H, brs), 5.96 (1H, brs), H-6′], [5.95 (1H, d, *J* = 2.4

Hz), 5.90 (1H, d, *J* = 2.4 Hz), 5.85 (1H, d, *J* = 2.4 Hz), 5.80 (1H, d, *J* = 2.4 Hz), H-6,8], [4.93 (1H, brs), 4.81 (1H, brs), H-2′], [4.64 (1H, d, *J* = 7.9 Hz), 4.47 (1H, dd, *J* = 5.9, 2.0 Hz), H-4], [4.58 (1H, t, *J* = 9.6 Hz), 4.32 (1H, t, *J* = 9.8 Hz), H-3], [4.42 (1H, d, *J* = 9.6 Hz), 4.31 (1H, overlapped), H-2], [4.23 (1H, m), 4.06 (1H, m), H-3′], [2.93 (1H, dd, *J* = 16.8, 4.4 Hz), 2.89 (1H, dd, *J* = 17.2, 5.1 Hz), H-4′α], [2.83 (1H, m), 2.71 (1H, dd, *J* = 17.2, 2.1), H-4′β]. ^{13}C-NMR (CD_3OD, 125 MHz) δ 158.8, 158.6 (C-8a), 157.6, 157.4, 157.3 (C-5,7), 156.5, 156.4, 156.0, 155.9, 155.5 (C-5′,7′,8′a), 146.6, 146.2, 146.1, 146.0, 145.7, 145.6 (C-11,11′,12,12′), 132.7, 132.3 (C-9), 132.5, 131.8 (C-9′), 121.3, 120.6 (C-14), 120.4, 119.2 (C-14′), 116.5, 116.4 (C-10), 116.2 (C-13), 116.1, 116.0 (C-13′), 115.3, 114.9 (C-10′), 108.8, 108.4 (C-8′), 107.5, 107.3 (C-4a), 101.6, 99.5 (C-4′a), 97.8, 97.7, 97.2, 96.2 (C-6,8), 97.6, 96.5 (C-6′), 84.1, 83.9 (C-2), 80.1, 80.0 (C-2′), 74.0, 73.9 (C-3), 67.9, 67.5 (C-3′), 38.9, 38.8 (C-4), 30.2, 29.4 (C-4′).

Kaempferol (10)

Yellow powder. $C_{15}H_{10}O_6$. m.p. 284 °C. IR (KBr) ν_{max} cm^{-1}: 3314, 1657, 1606, 1169. HR ESI-MS: *m/z* 309.0374 $[M + Na]^+$ (calcd. for 309.0375). ^{1}H-NMR (CD_3OD, 500 MHz): δ 8.08 (2H, d, *J* = 8.6 Hz, H-2′,6′), 6.90 (2H, d, *J* = 8.6 Hz, H-3′,5′), 6.39 (1H, s, H-8), 6.18 (1H, s, H-6). ^{13}C-NMR (CD_3OD, 125 MHz): δ 177.5 (C-4), 165.8 (C-7), 162.6 (C-5), 160.7 (C-4′), 158.4 (C-9), 148.2 (C-2), 137.2 (C-3), 130.8 (C-2′,6′), 123.8 (C-1′), 116.4 (C-3′,5′), 104.6 (C-10), 99.4 (C-6), 94.6 (C-8).

Astragalin (11)

Yellow powder. $C_{21}H_{20}O_{11}$. m.p. 218 °C. IR (KBr) ν_{max} cm^{-1}: 3362, 1656, 1607, 1505, 1447, 1282, 1179. HR ESI-MS: *m/z* 471.0900 $[M + Na]^+$ (calcd. for 471.0903). ^{1}H-NMR (CD_3OD, 500 MHz): δ 8.05 (2H, d, *J* = 8.8 Hz, H-2′,6′), 6.88 (2H, d, *J* = 8.8 Hz, H-3′,5′), 6.39 (1H, brs, H-8), 6.19 (1H, d, *J* = 1.6 Hz, H-6), 5.25 (1H, d, *J* = 7.4 Hz, H-1″), 3.69 (1H, dd, *J* = 11.9, 2.3 Hz, H-6″), 3.53 (1H, dd, *J* = 11.9, 5.5 Hz, H-6″), 3.45 (1H, t, *J* = 9.1 Hz, H-2″), 3.42 (1H, t, *J* = 9.1 Hz, H-3″), 3.32 (1H, overlap, H-4″), 3.21 (1H, m, H-5″). ^{13}C-NMR (CD_3OD, 125 MHz): δ 179.6 (C-4), 166.2 (C-7), 163.2 (C-5), 161.7 (C-4′), 159.2 (C-9), 158.6 (C-2), 135.6 (C-3), 132.4 (C-2′,6′), 122.9 (C-1′), 116.2 (C-3′,5′), 105.8 (C-10), 104.2 (C-1″), 100.0 (C-6), 94.9 (C-8), 78.5 (C-5″), 78.2 (C-3″), 75.8 (C-2″), 71.5 (C-4″), 62.7 (C-6″).

***Trans*-tiliroside (12)**

Yellowish powder. $C_{30}H_{26}O_{13}$. m.p. 262 °C. IR (KBr) ν_{max} cm^{-1}: 3461, 1684, 1607, 1501, 1419, 1362, 1296, 1182. HR ESI-MS: *m/z* 617.1273 $[M + Na]^+$ (calcd. for 617.1271). ^{1}H-NMR (CD_3OD, 500 MHz): δ 7.99 (2H, d, *J* = 9.0 Hz, H-2′,6′), 7.40 (1H, d, *J* = 15.9 Hz, H-7‴), 7.30 (2H, d, *J* = 8.6 Hz, H-2‴,6‴), 6.81 (2H, d, *J* = 9.0 Hz, H-3′,5′), 6.79 (2H, d, *J* = 8.6 Hz, H-3‴,5‴), 6.30 (1H, d, *J* = 2.1 Hz, H-8), 6.13 (1H, d, *J* = 2.1 Hz, H-6), 6.07 (1H, d, *J* = 15.9 Hz, H-8‴), 5.24 (1H, d, *J* = 7.5 Hz, H-1″), 4.30 (1H, dd, *J* = 11.8, 2.2 Hz, H-6β″), 4.19 (1H, dd, *J* = 11.8, 6.7 Hz, H-6α″), 3.48 (1H, m, H-2″), 3.46 (1H, m, H-5″), 3.44 (1H, m, H-3″), 3.33 (1H, m, H-4″). ^{13}C-NMR (CD_3OD, 125 MHz): δ 179.6 (C-4), 168.9 (C-9‴), 166.2 (C-7), 163.1 (C-5), 161.7 (C-4′), 161.3 (C-4‴), 159.5 (C-2), 158.6 (C-9), 146.7 (C-7‴), 135.3 (C-3), 132.3 (C-2′,6′), 131.3 (C-2‴,6‴), 127.2 (C-1‴), 122.9 (C-1′), 116.9 (C-3‴,5‴), 116.2 (C-3′,5′), 114.9 (C-8‴), 105.7 (C-10), 104.1 (C-1′), 100.2 (C-6), 95.0 (C-8), 78.2 (C-3″), 76.0 (C-5″), 75.9 (C-2″), 71.9 (C-4″), 64.4 (C-6″).

Helichrysoside (13)

Yellowish powder. $C_{30}H_{26}O_{14}$. HR ESI-MS: *m/z* 633.1216 $[M + Na]^+$ (calcd. for 633.1220). ^{1}H-NMR (CD_3OD, 500 MHz): δ 7.59 (1H, brs, H-2′), 7.57 (1H, d, *J* = 8.2 Hz, H-6′), 7.39 (1H, d, *J* = 15.9 Hz, H-7‴), 7.31 (2H, d, *J* = 8.1 Hz, H-2‴,6‴), 6.80 (1H, d, *J* = 8.2 Hz, H-5′), 6.79 (2H, d, *J* = 8.1 Hz, H-3‴,5‴), 6.30 (1H, brs, H-8), 6.13 (1H, brs, H-6), 6.08 (1H, d, *J* = 15.9 Hz, H-8‴), 5.26 (1H, d, *J* = 7.4 Hz, H-1″), 4.31 (1H, brd, *J* = 11.6 Hz, H-6″a), 4.20 (1H, dd, *J* = 11.6, 6.6 Hz, H-6″b), 3.55–3.32 (4H, m, H-2″,3″,4″,5″). ^{13}C-NMR (CD_3OD, 125 MHz): δ 179.5 (C-4), 169.0 (C-9‴), 166.1 (C-7), 163.1 (C-5), 161.2 (C-4‴), 159.3 (C-2), 158.6 (C-9), 150.0 (C-4′), 146.6 (C-7‴), 146.0 (C-3′), 135.3 (C-3), 131.3 (C-2‴,6‴), 127.2 (C-1‴), 123.4 (C-6′), 123.2 (C-1′), 117.4 (C-2′), 116.9 (C-3‴,5‴), 116.0 (C-5′), 114.9 (C-8‴), 105.6 (C-10), 103.9 (C-1″), 100.1 (C-6), 94.9 (C-8), 78.1 (C-3″), 76.0 (C-5″), 75.8 (C-2″), 71.8 (C-4″), 64.4 (C-6″).

Betulinic acid-3-*O*-*trans*-caffeate (14)

White amorphous powder. $C_{39}H_{54}O_6$. m.p. 270 °C. IR (KBr) ν_{max} cm^{-1}: 3392, 2944, 1688, 1635, 1604, 1515, 1450, 1376, 1273, 1182, 1111, 978. HR ESI-MS: *m/z* 641.3813 $[M + Na]^+$ (calcd. for 641.3818). ^{1}H-NMR (CD_3COCD_3, 500 MHz): δ 7.53 (1H, d, *J* = 15.9 Hz, H-7′), 7.15 (1H, brs, H-2′), 7.03 (1H, d, *J* = 8.1 Hz, H-6′), 6.86 (1H, d, *J* = 8.1 Hz, H-5′), 6.29 (1H, d, *J* = 15.9 Hz, H-8′), 4.72 (1H, brs, H-29), 4.59 (1H, brs, H-29), 4.56 (1H, dd, *J* = 10.8, 5.3 Hz, H-3), 3.05 (1H, m, H-19), 2.36 (1H, m, H-13), 2.26 (1H, brd, *J* = 12.6 Hz, H-16), 1.91 (1H, m, H-22), 1.74 (1H, m, H-12), 1.73 (1H, m, H-1), 1.72 (2H, m, H-2), 1.70 (3H, s, CH_3-30), 1.65 (1H, m, H-18), 1.58 (1H, m, H-15), 1.57 (1H, m, H-6), 1.52 (1H, m, H-7), 1.50 (1H, m, H-22), 1.47 (2H, m, H-6,16), 1.46 (1H, m, H-11), 1.45 (1H, m, H-9), 1.40 (2H, m, H-21), 1.39 (1H, m, H-7), 1.27 (1H, m, H-11), 1.20 (1H, m, H-15), 1.11 (1H, m, H-12), 1.08 (1H, m, H-1), 1.04 (3H, s, CH_3-27), 0.97 (3H, s, CH_3-26), 0.93 (3H, s, CH_3-24), 0.92 (1H, m, H-5), 0.91 (3H, s, CH_3-25), 0.88 (3H, s, CH_3-23). ^{13}C-NMR (CD_3COCD_3, 125 MHz): δ 178.0 (C-28), 167.6 (C-9′), 152.0 (C-20), 149.2 (C-4′), 146.8 (C-3′), 145.8 (C-7′), 128.1 (C-1′), 122.9 (C-6′), 116.8 (C-5′), 116.7 (C-8′), 115.6 (C-2′), 110.5 (C-29), 81.4 (C-3), 57.2 (C-17), 56.6 (C-5), 51.7 (C-9), 50.4 (C-18), 48.4 (C-19), 43.7 (C-14), 42.0 (C-8), 39.6 (C-1), 39.5 (C-13), 39.1 (C-10), 38.4 (C-4), 38.0 (C-22), 35.5 (C-7), 33.3 (C-16), 31.8 (C-21), 30.9 (C-15), 28.8 (C-23), 26.8 (C-12), 25.0 (C-2), 22.2 (C-11), 19.9 (C-30), 19.4 (C-6), 17.5 (C-24), 17.1 (C-25), 17.0 (C-26), 15.5 (C-27).

Ursolic acid-3-*O*-*trans*-caffeate (15)

White amorphous powder. $C_{39}H_{54}O_6$. m.p. >300 °C. IR (KBr) ν_{max} cm^{-1}: 3366, 2927, 1693, 1604, 1515, 1446, 1368, 1273, 1183, 1112, 977. HR ESI-MS: *m/z* 641.3816 $[M + Na]^+$ (calcd. for 641.3818). $[\alpha]_D^{22}$ +62.2 (*c*=0.1, MeOH). ^{1}H-NMR (CD_3COCD_3, 500 MHz): δ 7.53 (1H, d, *J* = 15.9 Hz, H-7′), 7.15 (1H, brs, H-2′), 7.04 (1H, d, *J* = 8.1 Hz, H-6′), 6.86 (1H, d, *J* = 8.1 Hz, H-5′), 6.29 (1H, d, *J* = 15.9 Hz, H-8′), 5.23 (1H, m, H-12), 4.58 (1H, dd, *J* = 11.0, 4.6 Hz, H-3), 2.24 (1H, d, *J* = 11.3 Hz, H-18), 1.16 (3H, s, CH_3-27), 1.01 (3H, s, CH_3-25), 0.96 (3H, d, *J* = 6.4 Hz, CH_3-30), 0.95 (3H, s, CH_3-24), 0.90 (3H, s, CH_3-23), 0.89 (3H, d, *J* = 6.7 Hz, CH_3-29), 0.85 (3H, s, CH_3-26). ^{13}C-NMR (CD_3COCD_3, 125 MHz): δ 178.8 (C-28), 167.2 (C-9′), 148.7 (C-4′), 146.4 (C-3′), 145.4 (C-7′), 139.4 (C-13), 127.8 (C-1′), 126.2 (C-12), 122.5 (C-6′), 116.4 (C-5′), 116.3 (C-8′), 115.3 (C-2′), 81.0 (C-3), 56.1 (C-5), 53.9 (C-18), 48.4 (C-9), 48.3 (C-17), 43.0 (C-14), 40.5 (C-8), 39.9 (C-19, 20), 39.1 (C-1), 38.6 (C-4), 37.8 (C-10), 37.7 (C-22), 33.9 (C-7), 31.4 (C-21), 28.9 (C-15), 28.6 (C-23), 25.0 (C-16), 24.5 (C-2), 24.1 (C-11), 24.0 (C-27), 21.5 (C-30), 19.0 (C-6), 17.7 (C-26), 17.6 (C-29), 17.4 (C-24), 16.0 (C-25).

1-mono(22-*O*-feruloyl-oxydocosanoyl)glycerol (16)

White amorphous powder. $C_{35}H_{58}O_8$. m.p. 104 °C. IR (KBr) ν_{max} cm^{-1}: 3511, 2918, 2849, 1723, 1636, 1600, 1518, 1467, 1269, 1174. HR ESI-MS: *m*/*z* 629.4027 $[M + Na]^+$ (calcd. for 629.4029). $[\alpha]_D^{25}$ +29.8 (*c*=0.05, $CHCl_3$). ^{1}H-NMR ($CDCl_3$, 500 MHz): δ 7.61 (1H, d, *J* = 15.9 Hz, H-7′), 7.07 (1H, brd, *J* = 8.2 Hz, H-6′), 7.04 (1H, brs, H-2′), 6.91 (1H, d, *J* = 8.2 Hz, H-5′), 6.29 (1H, d, *J* = 15.9 Hz, H-8′), 4.22 (1H, overlapped, H-1″), 4.19 (2H, overlapped, H-22), 4.15 (1H, overlapped, H-1″), 3.94 (1H, m, H-2″), 3.93 (3H, s, OCH_3), 3.70 (1H, dd, *J* = 11.4, 3.8 Hz, H-3″), 3.61 (1H, dd, *J* = 11.4, 5.8 Hz, H-3″), 2.34 (2H, t, *J* = 7.5 Hz, H-2), 1.70 (2H, m, H-21), 1.62 (2H, m, H-3), 1.40 (2H, m, H-20), 1.35–1.23 (30H, m, H-5~19), 1.29 (2H, overlapped, H-4). ^{13}C-NMR ($CDCl_3$, 125 MHz): 174.5 (C-1), 167.6 (C-9′), 148.1 (C-4′), 146.9 (C-3′), 144.8 (C-7′), 127.2 (C-1′), 123.2 (C-6′), 115.8 (C-8′), 114.9 (C-5′), 109.5 (C-2′), 70.4 (C-2″), 65.3 (C-1″), 64.8 (C-22), 63.5 (C-3″), 56.1 (C-OCH_3), 34.3 (C-2), 29.8–29.6 (C-5–17), 29.5 (C-18), 29.4 (C-19), 29.3 (C-4), 28.9 (C-21), 26.2 (C-20), 25.1 (C-3).
